# *Entamoeba muris* mitigates metabolic consequences of high-fat diet in mice

**DOI:** 10.1080/19490976.2024.2409210

**Published:** 2024-10-13

**Authors:** Maryline Roy, Anne Dumay, Sandrine Adiba, Sylvana Rozes, Seiki Kobayashi, Valérie Paradis, Catherine Postic, Dominique Rainteau, Eric Ogier-Denis, Maud Le Gall, Ulrich Meinzer, Emilie Viennois, Maite Casado-Bedmar, Alexis Mosca, Jean-Pierre Hugot

**Affiliations:** a Inflammation Research Centre, UMR 1149, INSERM, Université Paris Cité, Paris, France; b Paris Centre for Microbiome Medicine (PaCeMM) FHU, Paris, France; c Département de biologie, institut de Biologie de l’ENS, Ecole Normale Supérieure, CNRS, INSERM, Université PSL, Paris, France; d Department of pediatric gastroenterology and nutrition, Hôpital Robert Debré, Assistance Publique-Hôpitaux de Paris, Paris, France; e Department of Parasitology, National Institute of Infectious Diseases, Shinjuku-ku, Tokyo, Japan; f Department of pathology, Hôpital Beaujon, Assistance Publique-Hôpitaux de Paris, Clichy, France; g Institut Cochin, CNRS, INSERM, Université Paris Cité, Paris, France; h INSERM, Centre de Recherche Saint-Antoine, CRSA, AP-HP, Saint Antoine Hospital, Sorbonne Université, Paris, France

**Keywords:** Metabolic syndrome, amoebas, *Entamoeba muris*, hepatic steatosis, metabolic dysfunction-associated steatotic liver disease, dysbiosis, cAMP

## Abstract

Metabolic syndrome (MetS) is a cluster of several human conditions including abdominal obesity, hypertension, dyslipidemia, and hyperglycemia, all of which are risk factors of type 2 diabetes, cardiovascular disease, and metabolic dysfunction-associated steatotic liver disease (MASLD). Dietary pattern is a well-recognized MetS risk factor, but additional changes related to the modern Western life-style may also contribute to MetS. Here we hypothesize that the disappearance of amoebas in the gut plays a role in the emergence of MetS in association with dietary changes. Four groups of C57B/6J mice fed with a high-fat diet (HFD) or a normal diet (ND) were colonized or not with *Entamoeba muris*, a commensal amoeba. Seventy days after inoculation, cecal microbiota, and bile acid compositions were analyzed by high-throughput sequencing of 16S rDNA and mass spectrometry, respectively. Cytokine concentrations were measured in the gut, liver, and mesenteric fat looking for low-grade inflammation. The impact of HFD on liver metabolic dysfunction was explored by Oil Red O staining, triglycerides, cholesterol concentrations, and the expression of genes involved in β-oxidation and lipogenesis. Colonization with E. muris had a beneficial impact, with a reduction in dysbiosis, lower levels of fecal secondary bile acids, and an improvement in hepatic steatosis, arguing for a protective role of commensal amoebas in MetS and more specifically HFD-associated MASLD.

## Introduction

The development of the modern Western way of life is associated with an increasing prevalence of human morbid conditions including insulin resistance, obesity, metabolic dysfunction-associated steatotic liver disease (MSLD), dyslipidemia, and hypertension (altogether referred to as metabolic syndrome, or MetS). These conditions are among the main causes of type 2 diabetes and cardiovascular diseases which are themselves leading causes of death and disability in both developed and developing countries.^1,2^

The Western diet is a well-recognized risk factor for MetS (for review see).^3^ It is characterized by a high intake of saturated fats, refined grains, salt, and sugars, with a low consumption of fruits and vegetables. But the modern Western way of life has also influenced many other aspects of our lifestyle including hygiene, comfort, medicine, clothing, recreational activities, travels, and others, all of which are highly interconnected. Hence, other non-dietary MetS risk factors related to the Westernized lifestyle may have been neglected. Among these, we have here explored the potential impact of reduced exposure to intestinal eukaryotes.

Gut microbiota refers to the total amount of living organisms present in our digestive tract including bacteria, protozoa, fungi, or viruses. Bacteria are the best studied component. The Westernized diet is known to alter gut microbiota composition.^4^ A lower bacterial diversity and a reduced abundance of Firmicutes (newly renamed as Bacillota) are frequently reported.^5^ Among the best studied species with a beneficial impact, *Prevotella copri*, *Faecalibacterium prausnitzii*, *Alistipes* sp., and *Oscillobacter* sp. have been regularly reported while several *Clostridia* species, *Ruminococcus gnavus*, and *Flavonifractor plautii* are usually associated with a detrimental effect.^6–8^

The mammalian intestinal tract also hosts eukaryotes including macroparasites like helminths and single-celled organisms like protozoa.^9,10^ Eukaryotic microbes are generally considered to be parasites. This is the case for helminths and several protozoa like *Giardia lamblia, Entamoeba histolytica*, and *Cryptosporidium* spp., which contribute to well-defined diseases. In contrast, protozoa like *Blastocystis hominis, Dientamoeba fragilis*, or *Entamoeba* spp. (other than *E. histolytica*) exhibit no or limited pathogenicity.^10^ They are present across various healthy human populations worldwide, and recent studies have reported their higher prevalence in healthy controls compared to patients with digestive symptoms or diseases like inflammatory bowel disease.^10,11^ These findings, together with the co-evolution over hundreds of thousands of years between protozoa and their hosts,^12^ suggest that their interaction should be viewed as commensalism rather than parasitism.^13^ Hence, their recent disappearance from the human digestive tract of Westernized populations could be detrimental.

Protozoans are mainly present on vegetables and fruits as demonstrated by numerous reports (for review see)^14^ but they do not seem to be associated with fat intake.^12,15,16^ Among protozoa, *B. hominis* and *D. fragilis* remain fairly common in the Western world. Conversely, despite their worldwide distribution, *Entamoeba coli*, *E. dispar*, and *E. hartamni* have become rare in Westernized populations but remain prevalent in rural developing countries.^9,15,17^ Therefore, these commensal entamoebas are good candidates for investigating the role of protozoa on MetS. Unlike *E. histolytica*^18^, *Entamoeba coli*, and other commensal species have no obvious pathogenic effect.^10^ Conversely, they have been associated with beneficial changes in the gut bacterial component of the microbiome, including increased diversity and higher levels of bacteria belonging to the phylum Firmicutes (Bacillota).^15,19–22^ Consequently, they may have the potential to mitigate the effect of western diet.

To examine the hypothesis of the beneficial effect of intestinal colonization by commensal amoeba, we investigated the consequences of inoculating mice with *Entamoeba muris* (*E. muris)* on the microbiota and MetS-related parameters. This amoeba is closely related to *Entamoeba coli*, known to colonize the human digestive tract^23^ and it is recognized as nonpathogenic, even in immunocompromised animals raised under standard conditions.

## Methods

### E. muris: in vitro *culture, cyst purification, microscopic observation, and PCR methods*

*E. muris* was cultured *in vitro* and cryopreserved according to Kobayashi et al. ^23^ A detailed protocol is also available as supplementary information. Trophozoites and cysts were counted under a light microscope (x20 and x60 objectives, department of parasitology, Bichat hospital, Paris) at 24-h intervals for 5 days. On day 5, cysts were centrifuged (5 min at 275 G), rinsed with distilled water, and treated with 0.05 N chloridric acid for 10 min in order to retain only cysts resistant to gastric passage. They were then counted and resuspended in water at a concentration of 1,000 cysts/100 μl before inoculation. During follow-up and at the end of the experiments, microscopic observation of *E. muris* was performed, respectively, in the feces and cecal content of mice. Merthiolate, Iodine, Formol (MIF) staining was used to better visualize cytoplasmic and nuclear structures.

The presence of *E. muris* was also monitored by polymerase chain reaction (PCR) and real-time PCR. In brief, DNA was extracted from mouse feces frozen at −80°C using the QIAamp Fast DNA Stool Mini Kit, according to the manufacturer’s recommendations (Qiagen). Mechanical lysis with FastPrep (MP Biomedicals) was added to the protocol prior to thermal lysis at 95°C. Specific amplification of *E. muris* 18S RNA gene region was performed using the reported primers. (suppl. Table S1). Real-time polymerase chain reaction was carried out with SYBR Green (Qiagen) using the LightCycler® 480 (Roche). Relative DNA concentrations were reported according to the Δct method.

### Mouse experiments and analytical procedures

Preliminary experiments were conducted on a small group of three males and four females C57Bl/6J mice. We failed to identify significant differences between sex for amoeba infestation and microbiota composition (supplementary figure S1). We thus decided to perform the analyses on males only in order to limit differences in metabolic responses between sex.

Five-week-old wild-type (WT) C57Bl/6J male mice (Janvier, France) were acclimatized for 1-week prior to experiments and kept under Specific Opportunist Pathogen Free (SOPF) conditions. Experiments were approved by the institutional animal care and use committee (APAFIS#12939 -201,801,081,625,584, Paris, France). Mice were inoculated by gavage with 1,000 cysts/100 μl for the experimental group or with 100 μl of sterile water for the control group. The high-fat diet (SAFE®, 246 hF) was started in mice 9 days after gavage. The percentage of calories provided by lipids was 45.5%. The “normal diet” (ND) groups received a maintenance diet (suppl. table S2). For 70 days, the animals were weighed twice a week, food intake was recorded twice a week and fresh feces were collected and immediately frozen to study the microbiota and to monitor the presence of the amoeba by PCR every 2 weeks. After 70 days, mice were euthanized by cervical dislocation. Blood and tissue samples were collected for further analysis, as detailed below.

A first exploratory study (study 1) was performed to identify key changes associated with amoeba colonization. A replication study (study 2) was then performed to reproduce the most significant findings and to further document them by more focused analyses. Study 1 contained four groups of eight mice (ND (respectively HFD) with or without *E. muris*). As shown in the result section, we observed limited changes in the ND+*E. muris* group compared to the ND group. Thus, in order to reduce the number of animals (and to follow the recommendations of the French national ethic committee) this group of mice was not included in the replication study. Hence, only three groups remained in study 2 (six mice in the ND group and nine mice in each HFD group). As a result, the “ND with *E. muris”* group is underrepresented in figures showing combined datasets or absent for experiments made in study 2 only. For transparency, all available data are presented in the result section with no omission with a mention on the corresponding studies in figure legends. A schematic representation of the study design is available in supplementary figure S2.

Glucose tolerance tests were performed 2 weeks before the end of the experiments.^24^ Mice were gavaged with glucose at a dose of 2.0 g/kg body weight at 9 am, after 16 h of fasting. Tail blood glucose concentrations were measured at 0, 5, 15, 30, 60, 90, and 120 min. One week later, mice were intraperitoneally injected with insulin (ITT) at a dose of 1 U/kg body weight at 1 pm after 4 hours of fasting. Tail blood glucose concentrations were measured at 0, 5, 15, 30, 60, 90, and 120 min.

Blood glucose values were determined using an ACCU-Check glucose monitor (Roche Diagnostic Inc.). Serum high-density lipoprotein (HDL) cholesterol, triglycerides (TG), alanine aminotransferase (ALAT), and aspartate aminotransferase (ASAT) concentrations were determined using an automated Monarch device (Biochemistry Laboratory, Faculté de Médecine Bichat, France). Hepatic TGs and cholesterol were extracted with the Folch procedure and measured with a colorimetric kit according to the manufacturer’s instructions (Diasys).

Samples of the intestine and liver tissues were fixed in 4% paraformaldehyde, embedded in paraffin, cut, and stained with hematoxylin and eosin (H&E). Slides were assessed blind by an experienced pathologist. To detect fat deposition in the liver, frozen sections were immersed in isopropyl alcohol (VWR; 20839–297, 60% , 1 min), then in Oil Red O solution (DIAPATH; C0512, 10 min) and again in isopropyl alcohol (60%, 30 s). Thereafter, slides were rinsed with tap water, stained in hemalun Mayer (RAL diagnostics; 320550, 1 min) and rinsed again. At the end, they were scanned (ScanScope AT turbo®, Leica) at × 20 magnification and positive pixels were quantified by algorithm-positive pixels (Indica Labs, Albuquerque).

### 16S rRNA microbiome sequencing

DNA was extracted from cecal samples using a QIAamp PowerFecalPro DNA kit (Qiagen, Hilden, MD) following manufacturer’s instructions. Twenty-two DNA extracts were quantified on a Qubit4 fluorimeter using the dsDNA HS Assay Kit (Life Technologies; USA) and stored at −20°C before further analyses. DNA samples were sequenced after a two-step PCR library preparation, according to the recommendations of the 16S Metagenomic Sequencing Library Preparation Guide (Illumina, San Diego, CA, USA). Briefly, for the first PCR reaction, the V4 hyper-variable region of the 16S rDNA was amplified using the primers 515-F: 5’- TCG GCA GCG TCA GAT GTG TAT AAG AGA CAG GTG CCA GCM GCC GCG GTAA −3’ and 806-R: 5’- GTC TCG TGG GCT CGG AGA TGT GTA TAA GAG ACA GGG ACT ACH VGG GTW TCT AAT-3’. Amplicons were then purified using AMPure XP beads (Beckman Coulter, Indianapolis, IN). A second PCR reaction was performed to incorporate a sample-specific barcode, using a Nextera XT index kit (Illumina, USA). After amplicon purification, DNA concentration was controlled by qPCR using the KAPA Library Quantification kit (Roche). A master DNA pool was then generated in equimolar ratios. PhiX Control v3 (Illumina) was added to check the quality of the run. The pooled product quantity was controlled on a Qubit4 fluorimeter, loaded into an Illumina MiSeq cartridge, and sequenced on an IlluminaMiSeq sequencer (paired-end reads, 2 × 300 bp).

At the end of the run, FastQ files were generated and quality control was performed. Sequences were demultiplexed and paired-end amplicon reads were processed using the FROGS 3.2 pipeline (Find Rapidly OTU with Galaxy Solution), with the Galaxy platform (https://galaxy.migale.inra.fr/).^25^ Briefly, forward and reverse reads were trimmed for adaptor and PCR primers removal, merged, and chimeric sequences were removed. Reads were then clustered in operational taxonomic units (OTUs) and filtered with a minimum relative abundance threshold of 0.005%.

Taxonomic assignment was performed against the 16S SILVA 138 pintail 100 databases. Before analysis of alpha- and beta-diversities and microbiota composition, all samples were rarefied to the same depth, with a minimum read number of 25,000. Alpha-diversity within the group was estimated using the Shannon, Chao1, Simpson, and invSimpson diversity indexes. Beta-diversity between groups was evaluated by calculating distances between samples using the Bray-Curtis, Jaccard, Unifrac, and Weight-Unifrac methods. Ordination using principal coordinates analysis (PcoA) was performed to represent biodiversity distribution at the OTU level between groups. Lastly, differential biomarkers between various groups were observed using LEfSe algorithm (linear discriminant analysis coupled with effect size).^26^

### Bile acids analyses

In the feces, bile acids (BA) molecular species concentrations were measured by High-Performance Liquid Chromatography (HPLC) coupled to tandem mass spectrometry (HPLC-MS/MS) as previously described^27^ with slight modification. Two microliters of an internal standard solution (23-nor-5 β-cholanoic acid-3α, 12α-diol at 1 mg/ml) was added to 10–50 mg of feces lyophilized samples using a Lyovapor L200 (Buchi, Villebon-sur-Yvette, France). For 15–20 mg lyophilized feces samples, 2 ml of NaOH (0.1 M) was added and incubated for 1 h at 60°C before the addition of 4 ml of water. The solution was homogenized by two 10 s runs in an Ultra-Turrax disperser (IMLAB, Lille, France). The preanalysis cleanup procedure was achieved by centrifugation (12 000×*g* for 20 min) followed by solid-phase extraction using reversed-phase silica cartridges (HLB Oasis Waters) and we used a 5500Q-trap (Sciex) as mass spectrometer. The hydrophobicity index reflects BA hydrophobicity, taking into account the concentration and the retention time of different BAs on a C18 column with a gradient of methanol; lithocholic acid (LCA) has the highest retention time, and tauro-ursodeoxycholic acid-3S has the lowest. In the cecum, bile acid quantification was derived from metabolomic analyses.

### Untargeted metabolomics analysis

The analysis of metabolites was carried out by the pharmacology and immunoanalysis department (SPI) of the Joliot Institute at the CEA in Saclay using the LC-HRMS (Liquid Chromatography-High Resolution Mass Spectrometry). Ten mg aliquots of lyophilized cecal contents were used for the experiments. Untargeted metabolomics experiments were performed by LC-HRMS using a combination of two complementary chromatographic methods, consisting of reversed-phase chromatography (C18 chromatographic column) and hydrophilic interaction chromatography (HILIC) for the analysis of hydrophobic and polar metabolites, respectively. An internal standard solution was added to all the samples in order to check the consistency of the analytical results in terms of signal and retention time stability throughout the experiment. Additionally, a quality control sample was obtained by pooling 20 μl of each sample preparation. It was injected every 10 samples to evaluate the analysis error for each metabolite. Data extraction was performed by the platform. Compounds were annotated using public and internal databases (SPI). Mass spectrometry-based metabolite levels were calculated based on the area under the peak curve, which allows comparative analysis between groups but not absolute quantification. Detailed protocols are available as supplementary information.

### RNA isolation, quantitative real-time PCR assays, and cytokine analyses

Total RNAs were extracted from frozen tissues using NucleoSpin RNA kit according to manufacturer’s instructions (Macherey Nagel). RNA concentration and purity were determined by NanoDrop (Thermo Scientific). For reverse transcription of RNA into cDNA, High-Capacity cDNA Reverse Transcription Kit was employed. Real-time PCR was carried out with SYBR Green (Qiagen) using the LightCycler® 480 (Roche). Changes in mRNA expression were determined by calculating the fold-changes using the comparative threshold cycle (Ct) method using *Gapdh* as a house-keeping gene. Primer sequences are listed in Supplementary Table S3.

U-PLEX kits developed by the Mesoscale Discovery were used to assess the levels of some cytokines in the serum and tissues. Interleukin-1β (Il-1β), interleukin-6 (Il-6), interleukin-12 (Il-12), tumor necrosis factor⍺ Tnf-⍺, transforming growth factor β (Tgf-β), and interferon γ (Ifn-γ) were detectable in all tissues while interleukin-10 (Il-10) was not detectable in mesenteric fat. For blood analysis, serum was undiluted. For tissue analysis, proteins were extracted from frozen organs using an Ultra-Turrax and lysis buffer with protease and phosphatase inhibitors cocktail. Protein concentration was evaluated by Bradford protein assay (Bio-Rad). For Tgf-β, samples were acid-treated and base-neutralized prior to execution of the standard Mesoscale Discovery Soluble protein assessment protocols using the U-plex kit. Cytokine levels were measured from 25 μl of samples or standard. Plates were run on the Mesoscale Discovery instrument (Mesoscale Discovery) and cytokine levels were calculated using a standard curve of known cytokine quantities.

### Statistical analysis

GraphPad Prism 9 (GraphPad Software, La Jolla, CA) was used for statistical comparisons. All data are presented as mean ± SEM. Differences between the two groups were assessed by an unpaired two-tailed Student’s *t*-test (normal distribution) or by a two-tailed Mann–Whitney test (non-normal distribution). In the case of more than two groups, significance was analyzed by one-way ANOVA with Bonferroni multiple comparison test (normal distribution) or Kruskal–Wallis test with Dunn–Bonferroni test (non-normal distribution). For transparency, all performed statistical tests are reported in the figures with the mention “ns” for “not significant” when necessary. For statistical analysis of microbiota data, the significance of beta-diversity measures was assessed by the PERMANOVA (Permutational Multivariate Analysis of Variance Using Distance Matrices) test. For bacterial abundance, alpha-diversity and other data, ANOVA or the Kruskal–Wallis test, with multiple comparisons, were performed. A p-value <0.05 was considered significant (*p < .05; **p < .01; ***p < .001; ****p < .0001) for all analyses, with the exception of the LEfSE algorithm for which the p-value was set at 0.01.

## Results

### E. muris *can be cultured* in vitro,*its colonization maintained* in vivo, *and its presence detected by qPCR in the microbiota of colonized mice*

**Figure 1.**
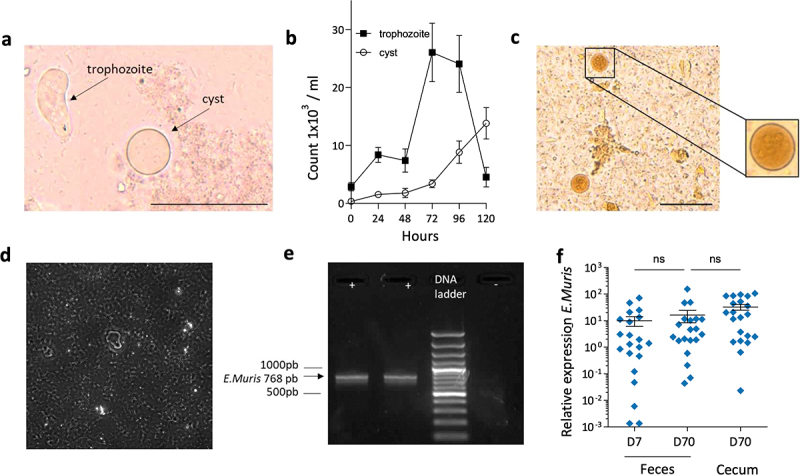
*In vitro* and *in vivo* characterization of *Entamoeba muris*. (a) Representative image and (b) cycle monitorization of cyst and trophozoite forms of *Entamoeba muris* cultured *in vitro*. (c) Representative image of the cyst form in a stool sample after Merthiolate iodine formaldehyde (MIF) staining. (d) video showing E.muris behavior is vailable at https://cri1149.fr/wp-content/uploads/2024/09/E.Muris2_.mp4. (e) Representative *Entamoeba* PCR identifying the presence of *E. muris* in three independent stool samples from negative control (-) and chronically colonized C57Bl/6J mice (+). (f) Relative expression of *E. muris* measured by Q-PCR in fecal and cecal samples at days 7 and 70 after infestation. Data are presented as mean ± SEM. Scale bars represent 50 μm (all studies including preliminary experiments).

*E. muris* was cultured *in vitro* according to the protocol previously described by Kobayashi et al. ^23^ The reproductive (trophozoites) and infective (cysts) forms of the amoeba harbored different shapes, bean, or round, respectively, so they could be clearly distinguished (Figure 1a). As expected, study of the population dynamics of these two stages revealed a shift in their relative abundances, with cysts increasing in prevalence while trophozoites decreased in parallel with the availability of nutrients in the medium (Figure 1b). Then, C57Bl/6J mice were inoculated with 1,000 cysts grown *in vitro*. Cysts were identified in the feces of these mice from day 7 after administration until the end of the experiment (Figure 1c). Trophozoites recovered from cecal samples were recognized as mobile forms of *E. muris* (see video Figure 1d).

To more easily and accurately detect the presence of amoebas, whatever their cystic or trophozoite forms, in feces and cecal contents, a qPCR based on *E. muris* sequence was developed (Figure 1e). This qPCR was further used throughout our study to monitor the presence of amoebas in colonized mice or their absence in control mice. Further, qPCR analyses showed stable amounts of *E. muris* 70 days after inoculation (Figure 1f) confirming the long-term colonization into the digestive tract. The amount of amoebas was not influenced by the diet (Figure S1d). We failed to see any notable clinical change concerning the aspect of feces, grooming, movement, or overall activity that would indicate symptomatic disease caused by *E.* *muris* in colonized mice.

### E. muris *partially restores the* Prevotellaceae/Desulfovibrionaceae *ratio in the microbiota of high-fat fed mice*

**Figure 2.**
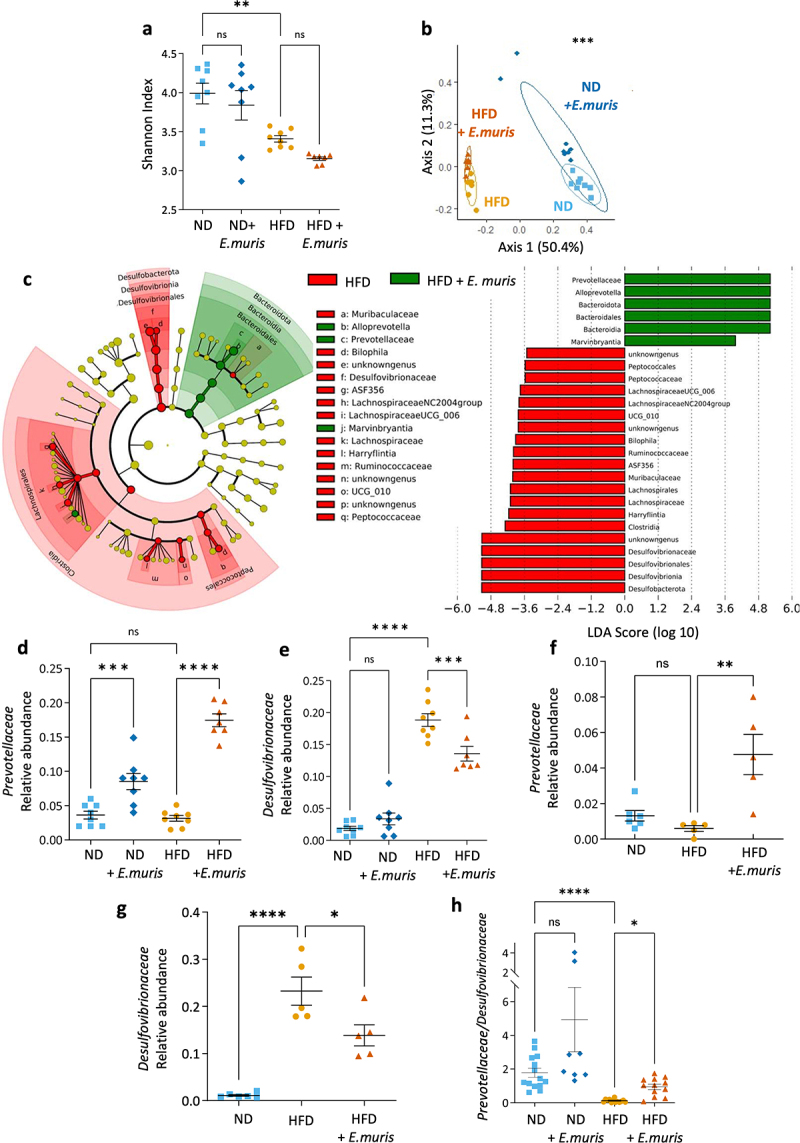
Colonization of *Entamoeba muris* is associated with a cecal dysbiosis characterized by more *prevotellaceae* and less *Desulfovibrionaceae*. At day 70, the cecal microbiota of C57Bl/6J mice fed with either normal (ND) or high-fat (HFD) diet and colonized with/out *Entamoeba muris* (*E. muris*) was analyzed. (a) Alpha-diversity is represented by the Shannon index for the four experimental groups (additional indexes are available in supplementary fig S3 and S4). (b) Principal coordinate analysis (PCoA) of the Bray Curtis matrix. Ellipses represent 95% of confidence. Statistical analyses were performed using PERMANOVA. (c) Taxonomic representation in a cladogram (left) and the linear discriminant analysis (LDA) score (right) of the of HFD mice groups in the presence (green) or absence (red) of *E. muris*. (d–g) relative abundance of the family *Prevotellaceae* and *Desulfovibrionaceae* in (d-e) the first experiment and (f–g) in the replication study. (h) *Prevotellaceae/Desulfovibrionaceae* ratio calculated for the pooled data. Dot plotted data presents mean ± SEM. Statistical analyses were performed using the one-way ANOVA test followed by a Bonferroni post hoc test. Significant differences were recorded as *p < .05, **p < .01, ***p < .001, ****p < .0001. Differences corresponding to *p* values lower than 0,01 are reported for LDA analyses. Data were generated during study 1 (a–e), study 2 (f–g) or both (h).

Since the amoeba is resident of the cecum,^28^ we focused on the changes in microbiota induced by *E. muris* in this organ. After a 70-day inoculation period, we compared four groups of eight mice fed either a normal (ND) or a high-fat diet (HFD) and colonized or not with *E. muris*. High-throughput sequencing of 16S rDNA revealed that diet had a much greater impact on the microbiota composition than the presence of the amoeba, both in terms of alpha- (Figure 2a) and beta-diversity (Figure 2b). Of note, we did not retrieve an increased number of the bacteria which were used for the dixenic culture of *E. muris* in colonized mice.

Although mice fed with ND and colonized with amoebas had no alterations of the alpha- or beta-diversities (Figure 2a,b and supplementary figure S3), their cecal microbial composition was altered, in particular with an increase in the *Alloprevotella* genus which pertains to the *Prevotellaceae* family (suppl. Figure S3). As expected, we observed a dysbiosis in HFD groups compared to ND groups, characterized by lower alpha-diversity (Figure 2a) and greater relative abundance of *desulfovibrionaceae* (suppl. Figure S3). In HFD-fed mice, the presence of *E. muris* significantly reduced some HFD-microbiota alterations (Figure 2c) by increasing *Prevotellaceae* (Figure 2d) and reducing *Desulfovibrionaceae* (Figure 2e). These findings were confirmed by a second independent experiment (Figure 2f,g and suppl. Figure S4). We therefore concluded that the presence of *E. muris* restored the *Prevotellaceae*/*Desulfovibrionaceae* ratio in the cecum of HFD-fed mice (Figure 2h).

### E. muris *partially restored HFD-induced changes in fecal bile acids*

**Figure 3.**
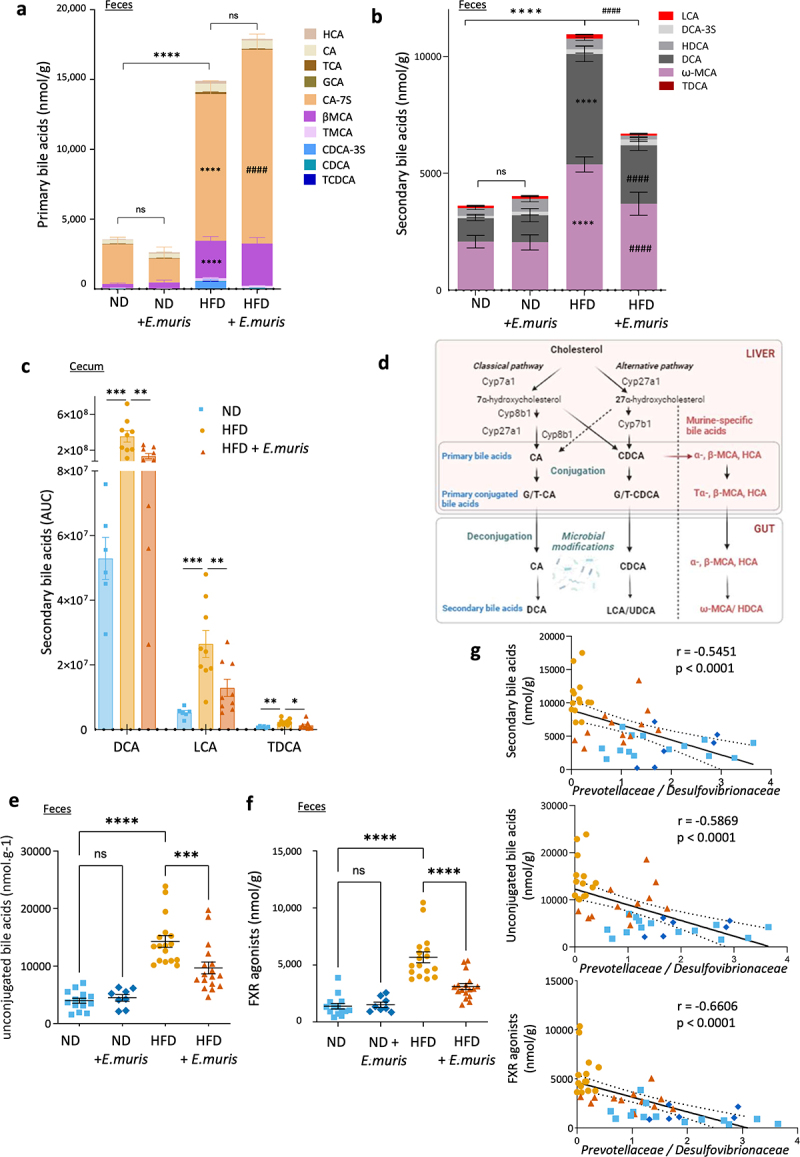
Cecal dysbiosis correlates with changes in bile acid composition. Bile acid composition was studied in either the feces or the cecum collected from C57Bl/6J mice fed with either normal diet (ND) or high-fat diet (HFD) and colonized with/out *Entamoeba muris* (*E. muris*). Abundance of primary (a) and secondary (b) BA in the feces. (c) relative abundance of secondary bile acids (DCA, LCA and TDCA) based on metabolomic analyses in the cecum of mice. (d) schematic representation of BA metabolism in the liver and the intestine. (e) abundance of total unconjugated bile acids and (f) farnesoid X receptor (FXR) agonist in the feces. (g) correlation analyses between the *Prevotellaceae/Desulfovibrionaceae* ratio and the fecal concentrations of secondary BA (top), unconjugated BA (middle), and FXR agonist (bottom). Data are presented as mean ± SEM, *n* = 8 - 18. Statistical analyses were performed using Mixed-effects analysis or one-way ANOVA test, both followed by a Bonferroni post hoc test. F-tests were used to determine the significance of the correlation. Significant differences were recorded as *p < .05, **p < .01, ***p < .001, ****p < .0001 when comparing the same bile acid type between groups, and ####p < 0.0001 when comparing different bile acids in one same condition. βMCA: β-Muricholic acid, CA: cholic acid, CA-7S: cholic acid-7-sulfate, CDCA: chenodeoxycholic acid, CDCA-3S: chenodeoxycholic acid-3-sulfate, DCA: deoxycholic acid, DCA-3S: deoxycholic acid-3-sulfate, GCA: glycocholic acid, HCA: hyocholic acid, HDCA: hyodeoxycholic acid, LCA: lithocholic acid, TCA: taurocholic acid, TCDCA: tauro-chenodeoxycholic acid, TDCA: tauro-deoxycholic acid, TMCA: tauro-muricholic acid, ω-MCA: ω-Muricholic acid. Analyses were done on the pooled dataset from studies 1 and 2 except for the cecum which is based on study 2 only.

Given the known impact of the microbiota on the composition of bile acids (BAs),^29^ we analyzed BAs concentrations in mice feces by mass spectrometry. Under ND, the presence of amoeba did not significantly alter primary (Figure 3a) nor secondary (Figure 3b) fecal BA concentrations. In line with previous studies,^29^ HFD increased the levels of primary and secondary BA (Figure 3a,b). While the presence of *E. muris* did not lead to changes in total BA levels, in HFD-fed mice, it significantly reduced the level of secondary BAs, including deoxycholic acid (DCA) and ω muricholic acid (ωMCA) (Figure 3b). Metabolomic analyses confirmed these findings in the cecum. Lower concentrations of LCA, DCA, and Tauro-deoxycholic acid (TDCA) were observed (Figure 3c), suggesting an overall effect on the three BA conversion pathways in the cecum (Figure 3d). The levels of unconjugated BAs, the deconjugation of which is also linked to the microbiota, were also reduced when HFD-fed mice were colonized with *E. muris* (Figure 3e and suppl. Figure S5). Finally, BAs capable of activating the farnesoid X receptor (FXR) (i.e., CDCA, CA, DCA, and LCA) were also reduced (Figure 3f). All these changes were correlated negatively with the *Prevotellaceae*/*Desulfovibrionaceae* ratio in the cecum arguing for a close relationship between the microbiota and the fecal BA composition (Figure 3g).

Taken together, these data suggested that the presence of amoebae has the potential to partially restore the levels of secondary and unconjugated BA altered by HFD.

### E. muris *reduces HFD-induced increase in hepatic il-1β*

**Figure 4.**
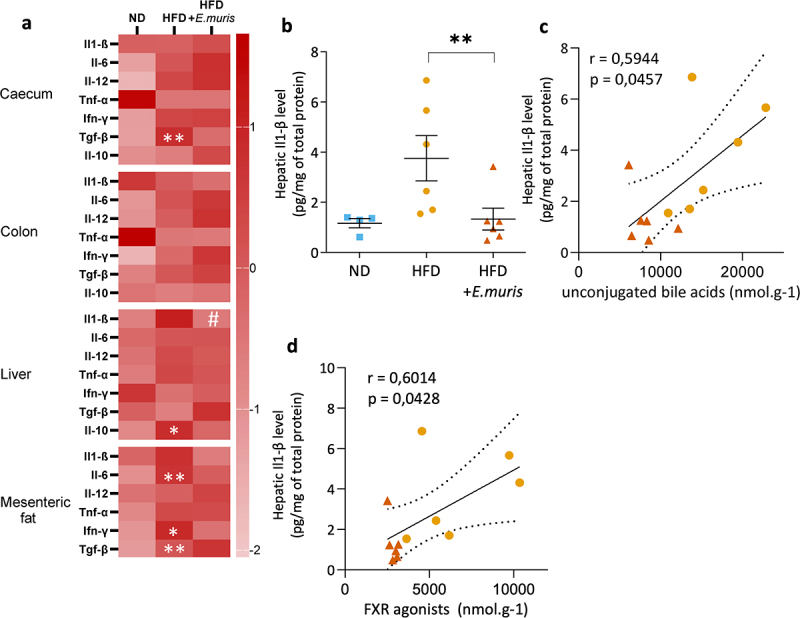
*Entamoeba muris* decreases IL-1β concentration in the liver. The concentrations of interleukin 1β (Il-1β), interleukin 6 (Il-6), interleukin 12 (Il-12), tumor necrosis factor ⍺ (Tnf-⍺), interferon γ (Ifn-γ), transforming growth factor beta (Tgf-β), and interleukin 10 (Il-10) were measured in the cecum, colon, liver, mesenteric fat of mice C57Bl/6J mice fed with either normal diet (ND) or high-fat diet (HFD) and colonized with/out *Entamoeba muris* (*E. muris*). (a) heatmap showing the cytokines levels (rows) that were significantly different between ND and HFD groups (*p < .05, **p < .01) or between HFD and HFD with E. muris groups (#p < 0.05). (b) Il-1β concentrations in the liver of the different experimental groups. (c–d) correlation analyses between Il-1β concentration and (c) unconjugated bile acids or (d) Farnesoid X receptor (FXR) agonist amounts in the feces. F-tests were used to determine the significance of the correlation. P-values are indicated in the corresponding figures. Measures were performed in study 2 mice. Il-10 was undetectable in mesenteric fat.

Unconjugated BAs have a known pro-inflammatory effect.^29^ Their lower concentration in HFD-fed mice colonized with *E. muris* suggested a potential protective effect against inflammation. To test this hypothesis, we assessed concentrations of pro- and anti-inflammatory cytokines, including Il-1β, Il-6, Il-10, Il-12, Tnf-α, Ifn-γ, and Tgf-β in the serum, cecum, colon, liver, and mesenteric adipose tissue. No significant changes were observed in the blood for any of these cytokines (suppl. Figure S6a). As expected, HFD-induced pro-inflammatory cytokines in several tissues (Figure 4a and suppl. Figure S6). Colonization with *E. muris* did not restore those changes with the exception of Il-1β levels in the liver (Figure 4b). Furthermore, hepatic Il-1β amounts strongly correlated with the presence of unconjugated BAs in the feces (Figure 4c). We also observed a correlation between Il-1β levels in the liver and the concentration of FXR-agonist BAs (Figure 4d). These results suggested that the presence of amoebae mitigated Il-1β secretion of HFD in the liver.

A low-grade gut inflammation has been regularly observed in HFD mice. We also identified few focal inflammatory lesions in the cecum and colon (but not in the small bowel) of HFD mice (suppl. Figure S7). These lesions were not seen in ND mice. Similar observations were made in HFD colonized with *E. muris* suggesting that amoeba colonization does not prevent the low-grade inflammation of the intestinal mucosa. This result was in accordance with cytokine quantifications in the large bowel.

### E. muris *reduces HFD-induced liver steatosis*

**Figure 5.**
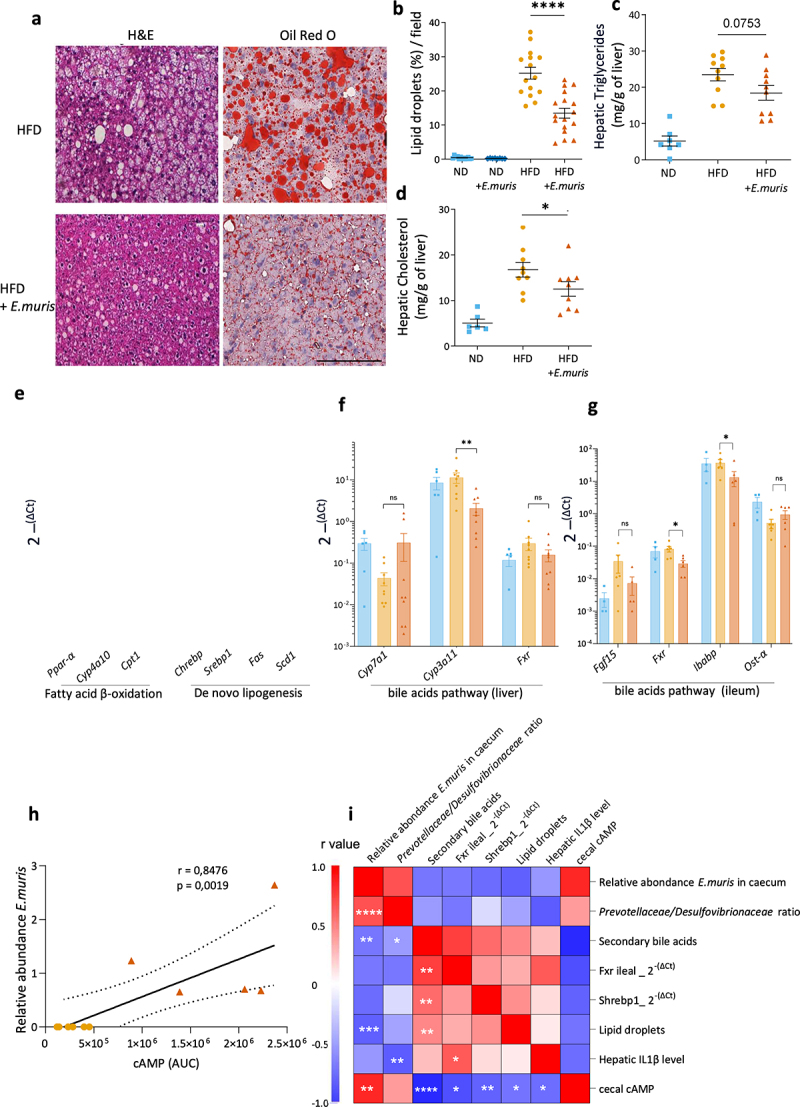
*Entamoeba muris* reduces hepatic steatosis. Study of the fatty liver disease in C57Bl/6J mice fed with normal diet (ND) or high-fat diet (HFD) and colonized with/out *eEntamoeba muris* (*E. muris*). (a) microscopic examination of hepatic steatosis after mouse livers were paraffin-embedded, sectioned, and stained with H&E and contrasted with oil red O for the visualization of lipids. Bar represents 100 μm. (b) percentages of the surface stained by oil red O as a marker of lipid accumulation. Hepatic (c) triglyceride and (d) cholesterol amounts in the liver. (e) mRNA expression levels of various genes related with the fatty acid β-oxidation and *de novo* lipogenesis. mRNA expression levels of various genes related with bile acid activating pathways in the liver (f) and ileum (g). (h) Correlation analysis between the relative abundance of *E. muris* and the concentration of cyclic adenosine monophosphate (cAMP) in the cecum. (g) heatmap showing the correlations between the different parameters modified by the presence of *E. muris* in HFD-fed mice by Spearman correlation. The color of each spot in the heatmap corresponds to the *r* value. Data are presented as mean ± SEM. Statistical analyses were performed using the Mann-wWhitney U test. Significant differences/correlations were recorded as *p < 0.05 and ****p < .0001. Data were provided by both studies 1 and 2 (a–b, i) or study 2 only (c–d, e–f).

Because HFD has been associated with MASLD, we examined the potential protective effect of amoeba in the liver tissue of HFD-fed mice. Hepatic steatosis, a consequence of HFD, was reduced in *E. muris* colonized mice compared with uncolonized mice (Figure 5a). Histological observations were confirmed by measuring the total surface of intracellular vacuoles (suppl. Figure S8a) and fatty areas stained with Oil Red O (Figure 5b). TGs (Figure 5c) and cholesterol (Figure 5d) concentrations in the liver were consistent with reduced fat accumulation even if they exhibited a border line statistical significance. Expression studies of genes involved in β-oxidation and lipogenesis suggested that the downregulation in lipogenesis, reflected by an average of two-fold lower expression of Chrebp (*p* = .06) and Srebp1 (*p* < .05), could explain the reduced accumulation of fat in the liver (Figure 5e). Notably, these changes were not associated with changes in blood levels of aspartate aminotransferase (ASAT) or alanine aminotransferase (ALAT) (suppl. Figure S8b and S8c).

Because liver cholesterol and TG metabolisms are regulated by *FXR* expressed in the small intestine and the liver, we looked for its expression and the expression of its downstream genes. We found a decreased expression of *Fxr* and *Ibabp* in small bowel suggesting that ileal Fxr pathway may be involved in the observed beneficial effect of amoeba colonization (Figure 5f,g).

Comparing Amoeba-colonized versus non-infested HFD-fed mice for other MetS components, we found a decreasing trend in circulating HDL cholesterol (suppl. Figure S8d), but this comparison did not reach statistical significance. A non-significant trend toward improvement was also observed for body weight gain (suppl. Figure S8e). Food intake was monitored all along the experiments without significant differences between HFD mice with/out *E.* *muris* (suppl. Figure S8f). No differences were seen for body fat composition (suppl. Figure S8g) including white fat (suppl. Figure S8h), and brown fat (suppl. Figure S8i) percentages. No differences were neither identified on glucose metabolism, either for fasting blood glucose (suppl. Figure S8j), orally induced hyperglycemia (suppl. Figure S8k), or blood glucose response to insulin (suppl. Figure S8l). Overall, in our hands, the main effect of the presence of *E. muris* on MetS was observed in the liver.

Lastly, metabolomic analyses performed in the cecum did not retrieve statistically significant changes except cAMP. cAMP was retrieved in colonized mice but not in control mice with a strong correlation between the cecal concentrations of cAMP and the relative abundance of *E. muris* (Figure 5h). At the end, a set of inter-related correlations was observed in HFD-fed mice linking *E. muris* abundance, cAMP concentration, cecal *Prevotellaceae*/*Desulfovibrionaceae* ratio, secondary fecal BA levels, *FXR* expression in the ileum together with Il-1β concentration, lipid accumulation, and lipogenesis in the liver (Figure 5i).

## Discussion

Our study on mice fed with HFD demonstrated that gut colonization by *E. muris* had a beneficial impact. It was characterized by a reduction in dysbiosis, changes in BA metabolism, reduced Il-1β secretion, and alleviation of hepatic steatosis. These results provide evidence for a protective role of amoebae in the context of MetS and more specifically MASLD. Of note, a limitation of this study is that we performed the experiments with males (in order to limit sex and hormonal differences previously reported).^30^ Preliminary data suggested that there are no major differences in term of amoeba colonization or microbiota composition between males and females, but our conclusions cannot be immediately extended to female mice.

In humans, HFD has consistently been associated with a reduction in microbial diversity and changes in microbiota composition.^31,32^ In accordance with the literature,^29,33,34^ our HFD mouse model presented a reduced microbial diversity, a decrease in *Prevotellaceae*, and an increase in *Desulfovibrionaceae*. *E. muris* colonization partially reversed the bacterial diet-induced dysbiosis by increasing *Prevotellaceae* and decreasing *Desulfovibrionaceae*. However, while previous studies have associated the presence of amoebae with greater richness and diversity of the bacterial microbiome in human,^15,19–22^ we did not observe such trends in our murine model. This difference may be related to the difficulty reestablishing microbiota diversity in laboratory mice due to highly stereotyped rearing conditions in specific pathogen-free facilities. Additional research involving rewilded mice could offer valuable insights.^35^

The impact of *E. muris* on the microbiome may explain its beneficial role on MetS. *Prevotellaceae* are known to dominate the gut microbiome of rural populations with a pre-industrial lifestyle while a decreased prevalence of *Prevotella* spp. in Westernized populations is compensated by *Bacteroides* spp.^36^ A *Prevotella*-rich gut microbiome improves weight loss, cholesterol levels, and glucose metabolism,^8,37^ especially in obese animals or under a Western diet.^38,39^ These beneficial effects are likely due to the potential of *Prevotella* spp. to degrade complex polysaccharides and to produce short-chain fatty acids, small molecules well-known for their beneficial impact on host health.^39^ A proliferation of *Desulfovibrio* spp. has been observed in HFD-fed animals suggesting their association with Western diets.^40^ Indeed, several studies have reported a positive correlation between *Desulfovibrio* spp. and MetS phenotypes including hypercholesterolemia, obesity, type 2 diabetes, and artery coronary disease in type 2 diabetes. Conversely, the abundance of *Desulfovibrio* spp. in type 2 diabetes models was reversed by different beneficial compounds derived from plants (for review see).^40^ Cecal *Desulfovibrio* abundance was also associated with obesity, dyslipidemia, and insulin resistance in pigs fed with a Western-style diet.^41^

However, if we observed a general trend toward improved MetS in HDF-fed animals colonized by *E. muris*, the impact of amoebas was only significant on the hepatic consequences of HFD. In line with our results, four studies reported a negative association between MASLD and *Prevotellaceae* in humans.^42–45^ To our knowledge, no study has reported a significant link between *Desulfovibrio* spp. and MASLD in humans.^46^ However, many works have reported a reduction in their abundance in association with the amelioration of HFD-induced MASLD in rodents.^40^ Conversely, gavage of HFD-fed mice with *Desulfovibrio piger* increased hepatic steatosis and fibrosis suggesting a causative role of this bacterium.^47^ The effects of *E. muris* on the microbiota are thus consistent with the observations made in MASLD.

Bacterial microbiota is known to impact hepatic functions via BA metabolism^48^ while, to our knowledge, nothing is known about a putative role of *E. muris* on BA metabolism. BAs are synthesized from cholesterol in the liver and then secreted into the gut. Most BAs are reabsorbed by the intestine and liver while only a small portion are excreted into feces. The gut microbiota converts conjugated BAs into unconjugated secondary BAs. Especially, Prevotella species have been recently reported to modulate BA metabolism in HFD mice.^49^ However, since neither *Prevotella* spp. nor *Desulfovibrio* spp. are known to be key deconjugation actors, the observed decreased deconjugation activity may be related to decreased abundances of alternative bacteria such as *Bacteroides*, which are negatively correlated with *Prevotella* abundance.

BAs modulate inflammation and tumorigenesis in the intestine, mesenteric fat, and liver through FXR signaling.^29^ *FXR* is mainly expressed in the intestine and in the liver where it plays a key role for glucose and lipid control (for review see).^50^ Under HFD, Fxr-deficient mice are usually protected against obesity and exhibit improved glucose homeostasis compared with control mice.^51^ Hepatic deletion of *Fxr* contributes to lipid accumulation in the liver *via* a *de novo* lipogenesis.^52^ HFD mice colonized by *E. muris* are characterized by a lower concentration of FXR agonist BAs and a decrease in *Fxr* expression in the ileum, in line with the effect of *E. muris* on hepatic steatosis.

Hepatic steatosis could thus be the consequence of hepatic *de novo* lipogenesis and increased TG storage. Our data argue for this hypothesis but future more in-depth experiments are necessary to firmly establish this point.^53^ Lipid accumulation results in lipotoxicity and inflammation *via* activation of Kupffer cells and macrophages. The activation could be related to the formation of cholesterol crystals with subsequent activation of the NOD-like receptor protein 3 (NLRP3)/Il-1β inflammasome pathway.^54^ However, diverse stimuli including mitochondrial damage, endoplasmic reticulum stress, and others may also activate the NLRP3 inflammasome and Il-1β secretion.^55^

Altogether, *E. muris* induces a comprehensive chain of effects in HFD-fed mice with improvements of the dysbiosis, BA metabolism, and signaling leading to less liver disease. But little is known about the inter-kingdom interactions between protozoa, bacteria, and the host which could initiate this proposed causative chain. Amoebae has a direct predatory effect on commensal bacteria. Less predation on species of the *Prevotella* genus and more predation on species of the *Desulfovibrio* genus could explain the observed changes. As demonstrated in several macro-ecosystems, indirect effects of predation could also explain the maintenance of greater microbiome biodiversity.^56^

An alternative explanation could be that amoebae generate or metabolize chemical components in the digestive tract that might affect bacterial growth and host response. Indeed, we found that the relative abundance of *E. muris* was correlated with cAMP level in the cecum of HFD-fed mice. cAMP is known to play a major role in the social organization of telluric amoebas (especially *Dictyostelium discoideum*). The self-organization of amoebas into aggregates, migrating slugs, and fruiting structures is under the control of cAMP signaling.^57^ cAMP in *Entamoeba* species is less studied but *E. histolytica* has a cAMP signaling system suggesting that this second messenger also plays a role in intestinal amoebaes.^58^ The correlation between amoebas’ abundance and cAMP concentration reported in the present study further points in this direction. Furthermore, cAMP has been shown to improve the impact of HFD on MetS in mice. Wang et al. reported that cAMP causes a reduction of the adipose tissue, a decreased size of adipocytes, decreased triglycerides and cholesterol levels in the serum, and finally less hepatic steatosis in high-fat-fed mice.^59^ The beneficial impact of amoebas in high-fat fed mice may thus also be directly related to cAMP production by amoeba.

To conclude, it should be noted that protozoa common to the Western world (mainly *Dientamoeba fragilis* and *Blastocystis hominis*) have very recently been associated with a lower prevalence of MetS in people with obesity.^8,12,60^ Here, we show in a mouse model that amoebas have a beneficial impact on MetS and more specifically on liver disease. A growing body of research therefore supports the idea that the disappearance of protozoa is a factor associated with modern Western lifestyle that contributes to the high incidence of MetS worldwide.

The recolonization of our digestive tract by commensal amoebas can thus be questioned. In theory, living with commensal protozoans could be seen as a normal condition with many benefits as living with our bacterial microbiota. However, amoebas suffer from a poor reputation: they are most often considered as putative pathogens (as it is the case for *E. histolytica*) even if no clear demonstration of pathogenicity has been done. But their reputation may change with safety data as it is the case for other eucaryotes present in our intestine like the yeast *Saccharomyces cerevisiae* or the protozoan *B. hominis*.^12^ Alternatively, the use of exogenous cAMP derivative products could constitute a less difficult approach for the treatment of MASLD and MetS.

## Supplementary Material

Supplemental Material

## Data Availability

All data generated or analyzed during this study are included in this published article.
