# Correction

**DOI:** 10.1080/19490976.2024.2421106

**Published:** 2024-10-28

**Article title**: Entamoeba muris mitigates metabolic consequences of high-fat diet in mice

**Authors**: Roy, M., Dumay, A., Adiba, S., Rozes, S., Kobayashi, S., Paradis, V., Postic, C., Rainteau, D., Ogier-Denis, E., Le Gall, M., Meinzer, U., Viennois, E., Casado-Bedmar, M., Mosca, A., and Hugot, J. P.

**Journal**: *KGMI: Gut Microbes*

**DOI**: https://doi.org/10.1080/19490976.2024.2409210

The article was originally published with the incorrect Figure 5.

**Figure 5.**
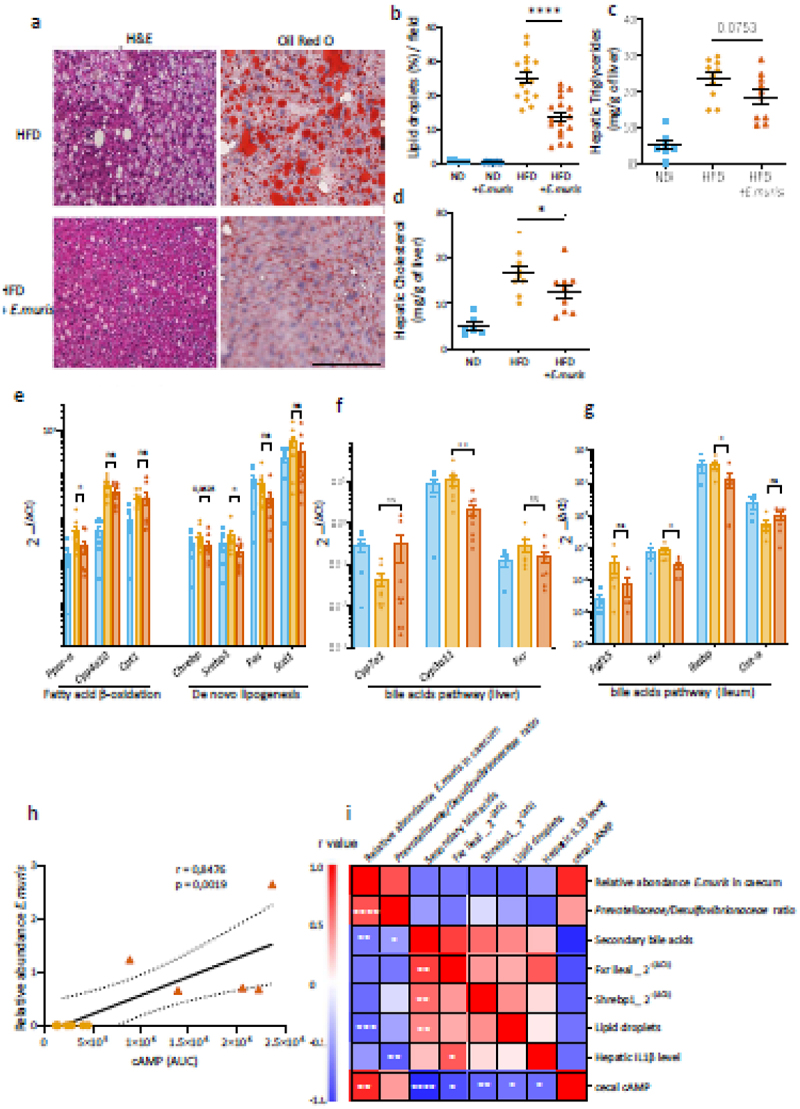
*Entamoeba muris* reduces hepatic steatosis. Study of the fatty liver disease in C57Bl/6J mice fed with normal diet (ND) or high-fat diet (HFD) and colonized with/out *eEntamoeba muris* (*E. muris*). (a) microscopic examination of hepatic steatosis after mouse livers were paraffin-embedded, sectioned, and stained with H&E and contrasted with oil red O for the visualization of lipids. Bar represents 100 μm. (b) percentages of the surface stained by oil red O as a marker of lipid accumulation. Hepatic (c) triglyceride and (d) cholesterol amounts in the liver. (e) mRNA expression levels of various genes related with the fatty acid β-oxidation and *de novo* lipogenesis. mRNA expression levels of various genes related with bile acid activating pathways in the liver (f) and ileum (g). (h) Correlation analysis between the relative abundance of *E. muris* and the concentration of cyclic adenosine monophosphate (cAMP) in the cecum. (g) heatmap showing the correlations between the different parameters modified by the presence of *E. muris* in HFD-fed mice by Spearman correlation. The color of each spot in the heatmap corresponds to the *r* value. Data are presented as mean ± SEM. Statistical analyses were performed using the Mann-wWhitney U test. Significant differences/correlations were recorded as *p < 0.05 and ****p < .0001. Data were provided by both studies 1 and 2 (a–b, i) or study 2 only (c–d, e–f).

The figure 5 provided below have been included in the original article, and it has been republished accordingly.

