# Supplementary material for: Entamoeba muris mitigates metabolic consequences of high-fat diet in mice
Source: Gut Microbes. 2024 Oct 13;16(1):2409210. doi: 10.1080/19490976.2024.2409210 (PMC11485694; doi:10.1080/19490976.2024.2409210)
Supplement: Supplemental Material [file KGMI_A_2409210_SM9895.docx]

Manuscript ID KGMI-S-2024-0491

"Entamoeba muris mitigates metabolic consequences of high-fat-diet in mice"

Supplementary information

**Supplementary Table 1.** Oligonucleotide primers used for PCR and QPCR assays of *Entamoeba muris*

| Assay | Forward sequence (5’- 3’) | Reverse sequence (5’- 3’) |
| --- | --- | --- |
| PCR_ *E.muris* | TGACTCAACACGGGAAAACTT | ATCCTTCCGCAGGTTCACCTAC |
| Q-PCR_ *E.muris* | ACACGCAAATGAAGCGTGAC | AACCCCGAAAGGAAAGAGGC |

**Supplementary Table 2.** Composition of mouse diets

| Diet | ND | |  | HFD | |
| --- | --- | --- | --- | --- | --- |
|  | % | Energy  (Kcal/kg diet) |  | % | Energy  (Kcal/kg diet) |
| Fat | 11 | 367 |  | 45,5 | 2263,8 |
| Protein | 24 | 768 |  | 15,9 | 791,9 |
| Carbohydrate | 65 | 2091 |  | 38,6 | 1917,7 |
| Total | 100 | 3326 |  | 100 | 4973,4 |

ND, nomal diet; HF, high-fat diet.

**Supplementary Table 3.** Oligonucleotide primers used for quantitative real-time RT-PCR analysis

| Gene | Forward sequence (5’- 3’) | Reverse sequence (5’- 3’) |
| --- | --- | --- |
| *Ppar-α* | GGTACCACTACGGAGTTCACG | GCTCCGATCACACTTGTCGT |
| *Cyp4-a10*  *Cpt1*  *Chrebp*  *Srebp-1*  *Fas*  *Scd1*  *Gapdh*  *Fxr*  *Fgf15*  *Ostb*  *Ibabp* | CAACACATCTCCTTAATGACCCTAGAC  TCTGGCAGTCGACTCACCTT  ATGACCCCTCACTCAGGGAATA  GACCCTTCCAGGAAACACTC  TTCCAAGACGAAAATGATGC  CCGGAGACCCCTTAGATCGA  CACCATCTTCCAGGAGCGAG  GCTTGATGTGCTACAAAAGCTG  ACGGGCTGATTCGCTACTC  GAGCATCCTGGCAAACAGA  GGCAAAGAATGTGAAATGCAG | CCTGTAATTTCCATCTACCTGAACACT  GCCACAGGACACATAGTCAGG  GATCCAAGGGTCCAGAGCAG  TGTTTGTTCTAGGGGCTGC  AATTGTGGGATCAGGAGAGC  TAGCCTGTAAAAGATTTCTGCAAA  GCCTTCTCCATGGTGGTGAA  CGTGGTGATGGTTGAATGTCC  TGTAGCCTAAACAGTCCATTTCCT  TGCAGGTCTTCTGGTGTTTCT  CCGAAGTCTGGTGATAGTTGG |

Material and methods. Supplementary information.

1. Protocole for *E. muris* culture
2. Preparation of modified Balamuth medium

Mix 36g dehydrated egg yolk (see note) with an equal amount of water, add 125mL 0.8% sodium chloridesolution and mix well.

NOTE: Fresh egg yolks may be used in preparing this medium in the proportion of 4 hard-boiled yolks suspended in 125 mL of 0.8% saline.

1. Boil the mixture with *constant stirring (the presence of some glass beads in the flask is recommended) or steam it at 80°C for 20 min. Add water if necessary to restore the original volume. * Use a hot plate stirrer, glass flask and thermometer
2. Filter through a double layer of muslin or gauze and add 0.8% saline to restore the volume to 125mL.
3. Cool the liquid to below 10°C and filter if it is required to give a clear solution.
4. Add 125mL M/15 phosphate buffer pH 7.5*.

* KH_2_PO_4_ soln. (9.08g/L), 20.5 mL:

Na_2_HPO_4_.2H_2_O soln. (11.88g/L), 10.45 mL.

1. Autoclave the liquid (15lb, 20min): it may be stored at 4°C for several months.
2. Add 4% heat inactivated (56°C for 3 hours) adult bovine, sheep or pig serum into the egg yolk solution.
3. Dispense (aseptically) 4 mL aliqxuots in sterile pyrex glass culture tube (16 x 100mm) with screw cap (No.13).
4. Add a loopful of sterile powdered whole rice starch.
5. Inoculate 0.2mL of *Escherichia coli* / (e.g. Balamuth medium) cultured suspension into the culture tube and cultured at 25°C prior to the cultivation of *E. muris* at least 1day before its subculturing.
6. *Bacteroides fragilis* is cultured by liquid medium such as *GAM medium, prior to the cultivation of *E. muris*. * See subculture.
7. Subcultures

1) Inoculate 0.2-0.4mL of *Bacteroides fragilis* culture suspension into the medium just before subculturing of *E. muris*.

2) Inoculate 0.6-1mL of *E. muris* cultured suspension into the medium and incubated at 25°C or 35.5°C and at an *angle of 5-10°.

3) After confirming the growth of amoebae, do subculture like step 1-2. Usually, number of amoebae is reached stationary phase until 3-4 days after inoculation.

NOTE: After 2-3 days, cysts are also produced in the culture medium.

1. Metabolomic: raw experimental data acquisition.

### Chemicals and reagents

All analytical grade reference compounds were from Sigma (Saint Quentin Fallavier, France). The standard mixtures used for the external calibration of the MS instrument (Calmix-positive, for the positive ion mode, consisting of caffeine, L-methionyl-arginyl-phenylalanyl-alanine acetate, and Ultramark 1621, and Calmix-negative, for the negative ion mode, consisting of same mixture plus sodium dodecyl sulfate and sodium taurocholate) were from Thermo Fisher Scientific (Courtaboeuf, France). Acetonitrile (ACN) was from SDS (Peypin, France), formic acid from Merck (Briare-le-Canal, France), methanol from VWR Chemicals (Fontenay-sous-Bois, France) and deionized water from Biosolve chemicals (Dieuse, France).

### Metabolite extraction

About exactly 10 mg of lyophilized caeca content were weighed in a Precellys tube (ref CK14-2 mL, Bertin) and resuspended in 750µL Methanol:H20 (80:20, v/v). After vortexing, samples were lysed in a Precellys Device (6500rpm - 3*30sec, 4°C), then samples were left on ice for 90 minutes to obtain complete deproteinization. After centrifugation at 20000 g for 15 minutes at 4°C, two equal volumes of the supernatant (187.5 µL) were evaporated to dryness under a nitrogen stream at 30°C using a Turbovap (Caliper Life Science Inc., Roissy, France). Then, 100 µL of H2O:Acetonitrile (CAN) (95:5, v/v), with 0.1% formic acid or 10 mM ammonium carbonate pH10.5:CAN (40:60, v/v) were added to the residue to reconstitute the samples for RP and HILIC analyses, respectively. The tubes were vortexed again, incubated in an ultrasonic bath for 5 minutes, and centrifuged for another 10 minutes. A volume of 95μL of the supernatant was transferred to 0.2 mL vials. Internal standard solution (5 μL; mixture of 9 authentic chemical standards covering the mass range of interest: 13C-glucose, 15N-aspartate, ethylmalonic acid, amiloride, prednisone, metformin, atropine sulfate, colchicine, imipramine) was added to all samples in order to check for consistency of analytical results in terms of signal and retention time stability throughout the experiment. In addition, a quality control (QC) sample was obtained by pooling 20 μL of each sample preparation. It was injected every 10 samples in order to evaluate the analytical error for each metabolite. (Area were normalized according to the initial weight with the objective to compare the same content per samples)

### Liquid chromatography coupled to high-resolution mass spectrometry

The ultra-high performance liquid chromatographic (UHPLC) separation was performed on a Hypersil GOLD C8 1.9 µm, 2.1 mm x 150 mm column (RP) at 30°C (Thermo Fisher Scientific, les Ulis, France), and HPLC chromatographic separations were performed on a Sequant ZICpHILIC 5 µm, 2.1 x 150 mm (HILIC) at 15°C (Merck, Darmstadt, Germany). All chromatographic systems were equipped with an on-line prefilter (Thermo Fisher Scientific, Courtaboeuf, France). Experimental settings for each LC/MS condition are described below. Mobile phases for RP columns were 100% water in A and 100% ACN in B, both containing 0.1% formic acid. Regarding HILIC, phase A consisted of an aqueous buffer of 10 mM ammonium carbonate in water adjusted to pH 10.5 with ammonium hydroxide, whereas pure ACN was used as solvent B. Chromatographic elutions were achieved under gradient conditions as follows: (i) RP-based system: the flow rate was set at 500 µL/min. The elution consisted of an isocratic step of 2 minutes at 5% phase B, followed by a linear gradient from 5 to 100% of phase B for the next 11 minutes. These proportions were kept constant for 12.5 min before returning to 5% B for 4.5 min. (ii) HILIC-based system: the flow rate was 200 µL/min. Elution started with an isocratic step of 2 min at 80% B, followed by a linear gradient from 80 to 40% of phase B from 2 to 12 min. The chromatographic system was then rinsed for 5 min at 0% B, and the run ended with an equilibration step of 15 min (80% B). LC-MS analyses were performed using a U3000 liquid chromatography system coupled to an Exactive mass spectrometer from Thermo Fisher Scientific (Courtaboeuf, France) fitted with an electrospray source operated in the positive and negative ion modes. The software interface was Xcalibur (version 2.1) (Thermo Fisher Scientific, Courtaboeuf, France). The mass spectrometer was calibrated before each analysis in both ESI polarities using the manufacturer’s predefined methods and recommended calibration mixture provided by the manufacturer (external calibration). The Exactive mass spectrometer was operated with capillary voltage at -3 kV in the negative ionization mode and 5 kV in the positive ionization mode and a capillary temperature set at 280°C. The sheath gas pressure and the auxiliary gas pressure were set, respectively, at 60 and 10 arbitrary units with nitrogen gas. The mass resolution power of the analyzer was 50,000 m/?m, full width at half maximum (FWHM) at m/z 200, for singly charged ions. The detection was achieved from m/z 85 to 1000 for RP conditions in the positive ionization mode and from m/z 50 to 1000 for HILIC conditions in the negative ionization mode.

## Metabolomic: Data treatment

### Processing

All raw data were manually inspected using the Qualbrowser module of Xcalibur version 2.1 (Thermo Fisher Scientific, Courtaboeuf, France). Raw files were first of all converted to mzXML format using MSConvert software. Automatic peak detection and integration were performed using the XCMS software package ([W4M platform](https://galaxy.workflow4metabolomics.org), ref: F. Giacomoni et al. bioinformatics, 2014), which returned a data matrix containing m/z and retention time values of features together with their concentrations expressed in arbitrary units (i.e., areas of chromatographic peaks). XCMS features were thereafter filtered according to the following criteria: (i) the correlation between dilution factors of QC samples and areas of chromatographic peaks (filtered variables should exhibit coefficients of correlation above 0.7 in order to account for metabolites occurring at low concentrations and which are not detected anymore in the most diluted samples), (ii) repeatability (the coefficient of variations obtained for chromatographic peak areas of QC samples should be below 30%) and (iii) ratio of chromatographic peak area of biological to blank samples above a value of 3. Optionally, if necessary, chromatographic peak areas of each variable present in the XCMS peak lists were normalized using the LOESS algorithm in order to remove analytical drift induced by clogging of the ESI source observed in the course of analytical runs.

### Annotation

Features were annotated by matching their accurate measured masses ± 10 ppm with theoretical masses contained in biochemical and metabolomic databases by using an informatics tool developed in R language. The databases used were the Kyoto Encyclopedia of Genes and Genomes (KEGG) (Kanehisa M, Goto S. KEGG: kyoto encyclopedia of genes and genomes. Nucleic Acids Res 2000 Jan 1;28(1):27-30), the Human Metabolome Database (HMDB) (Wishart DS, Tzur D, Knox C, Eisner R, Guo AC, Young N, et al. HMDB: the Human Metabolome Database. Nucleic Acids Res 2007 Jan;35(Database issue):D521-D526) and METLIN (Smith CA, O’Maille G, Want EJ, Qin C, Trauger SA, Brandon TR, et al. METLIN: a metabolite mass spectral database. Ther Drug Monit 2005 Dec;27(6):747-751). Features were also annotated by our spectral database according to accurately measured masses and chromatographic retention times (Boudah S, Olivier MF, ros-Calt S, Oliveira L, Fenaille F, Tabet JC, et al. Annotation of the human serum metabolome by coupling three liquid chromatography methods to high-resolution mass spectrometry. J Chromatogr B Analyt Technol Biomed Life Sci 2014 Sep 1;966:34-47). To be identified, ions had to match at least 2 orthogonal criteria (accurately measured mass, isotopic pattern, MS/MS spectrum and retention time) to those of an authentic chemical standard analyzed under the same analytical conditions, as proposed by the Metabolomics Standards Initiative (Sumner LW, Amberg A, Barrett D, Beale MH, Beger R, Daykin CA, et al. Proposed minimum reporting standards for chemical analysis. Metabolomics 2007 Sep;3(3):211-221).

#### KHM annotation

This annotation is done using public data bases (KEGG, METLIN and HMDB), faciliting the annotation of unknown metabolites. However, it is not currently and is only based on mz.

#### SPI annotation

This annotation is done using SPI intern data bases and is based on comparison of mz and retention time from experimental data to referent molecules from SPI chimiotech. Compared to annotation by public databases, this system takes into account the fact that mz are not necessarily pseudomolecular ions, but can be isotopes, adducts or fragments. It also takes into account the different chromatographic systems used in the laboratory for retention times (HILIC, C18).


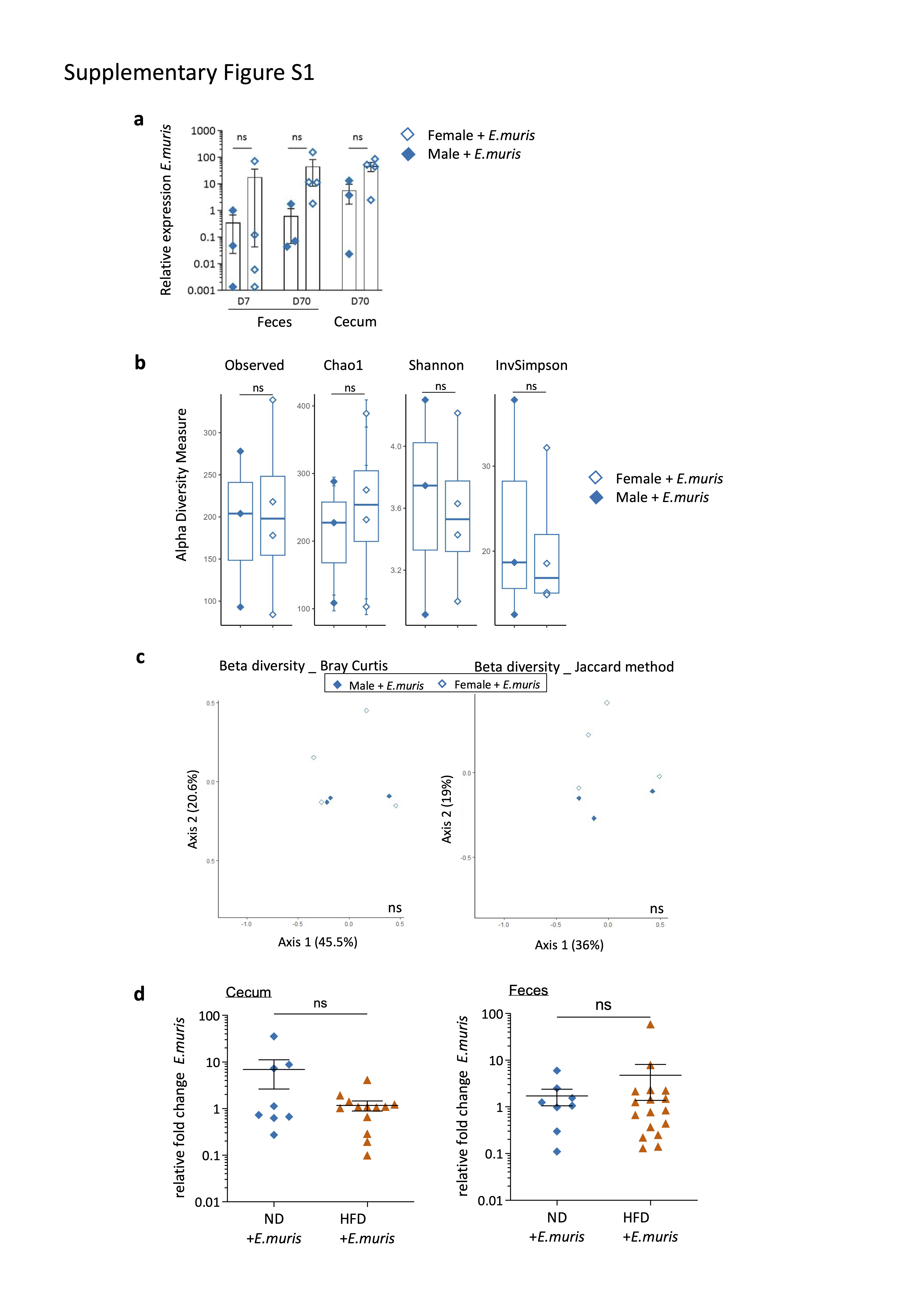


**Supplementary figure S1. Preliminary experiments exploring putative sex differences in infested mice and the impact of diet on amoeba abundance.** Mice under a normal diet were all infested by *E. muris* **(a)** Relative expression of *E. muris* measured by Q-PCR in the feces and cecum. (b) alpha-diversity and (c) beta-diversity measured by several indexes in the cecum. (d) Relative abundance of *E. muris* measured by Q-PCR in the caecum and feces of mice under normal (ND) or high fat (HFD) diet, 70 days after oral gavage of 1000 cysts.

**
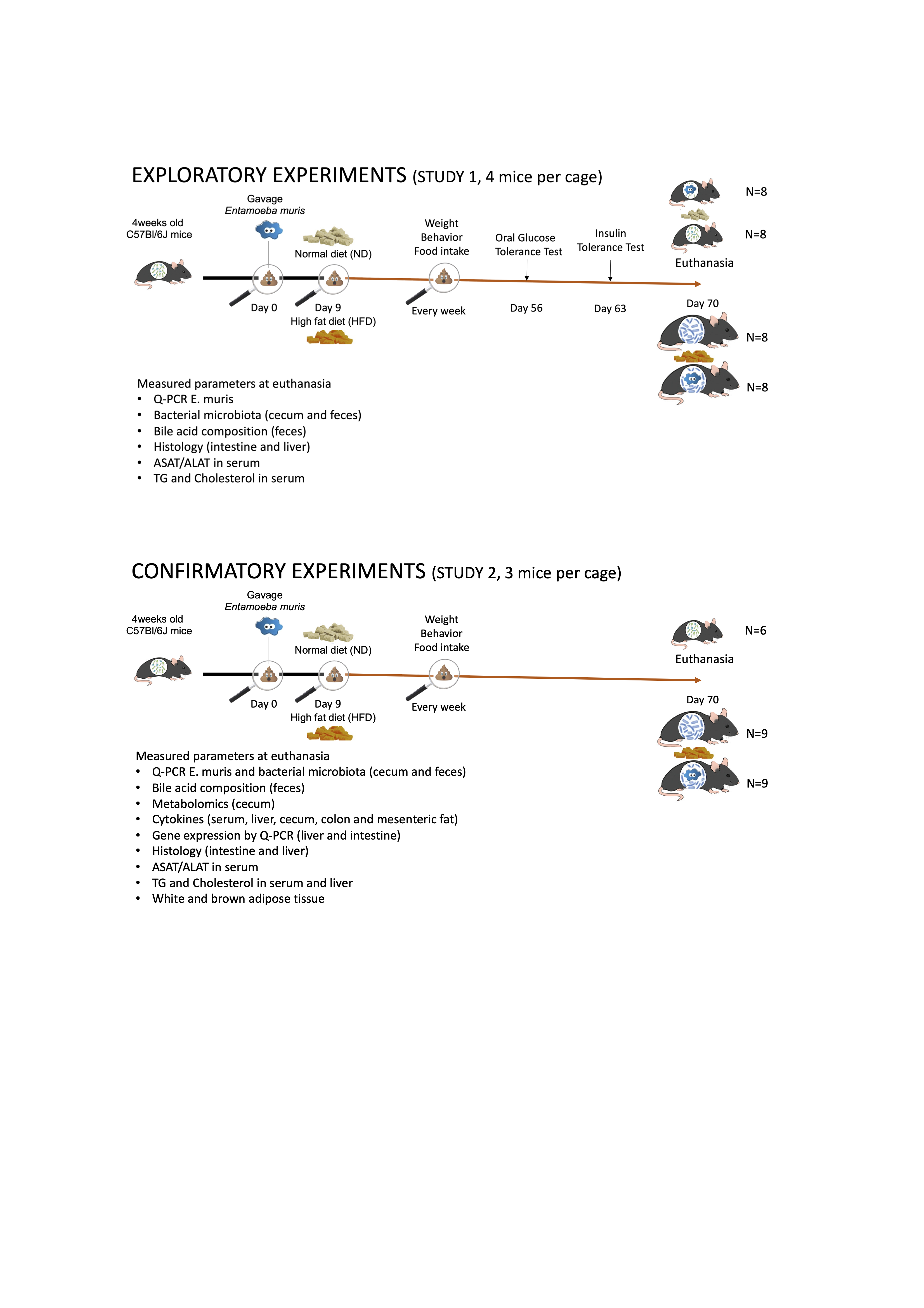
**

**Supplementary figure S2. Schematic representation of the experimental design.** Two independent sets of experiments were performed. The exploratory study 1 allowed to identify some parameters improved in HFD mice with *E. muris*. No major changes were seen between the ND groups with and without *E. muris*. Thus the “ND with amoebas” group was deleted in the confirmatory study 2. Different experiments were also proposed in study 2 to further explore the observed changes.**
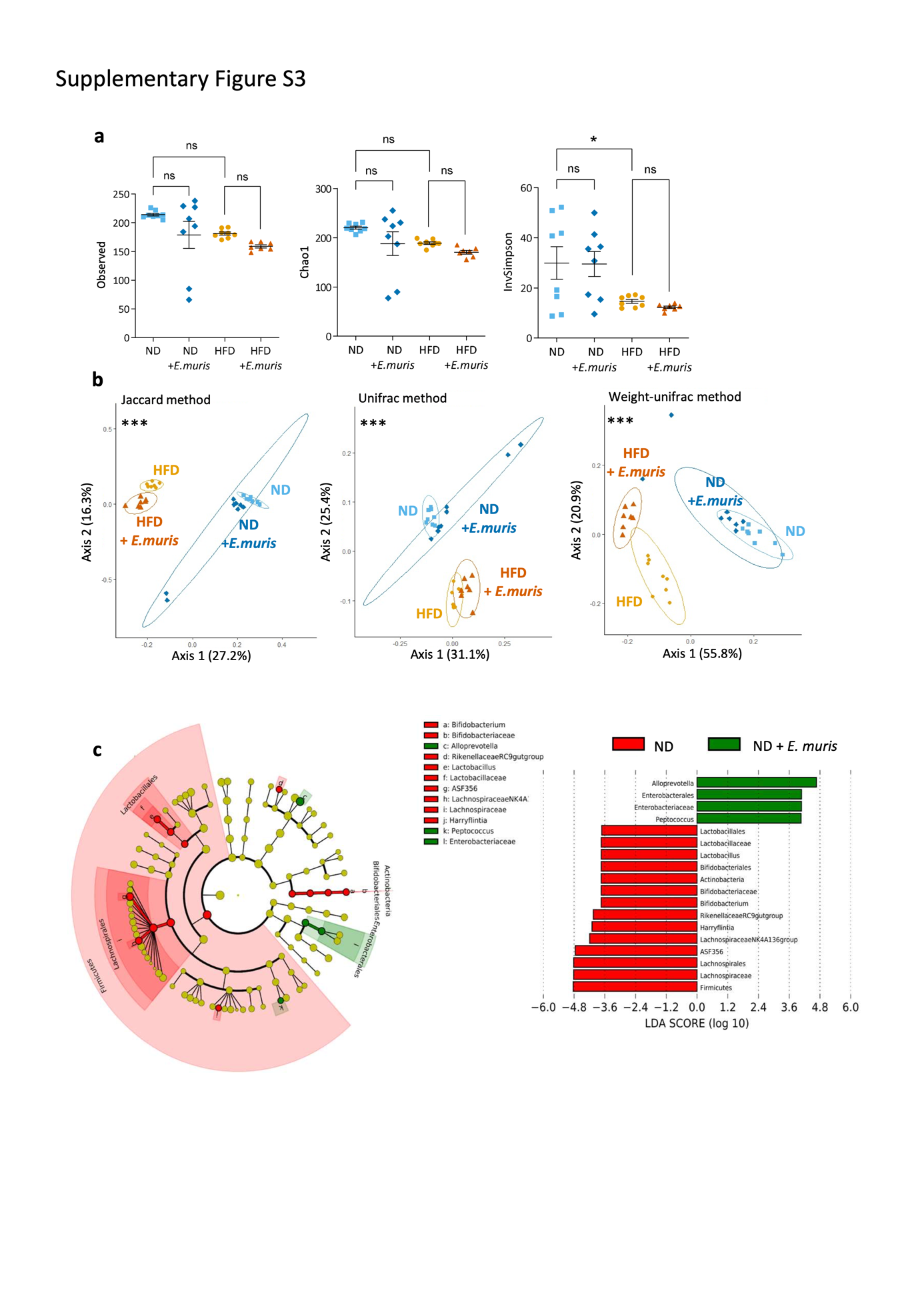
**

**
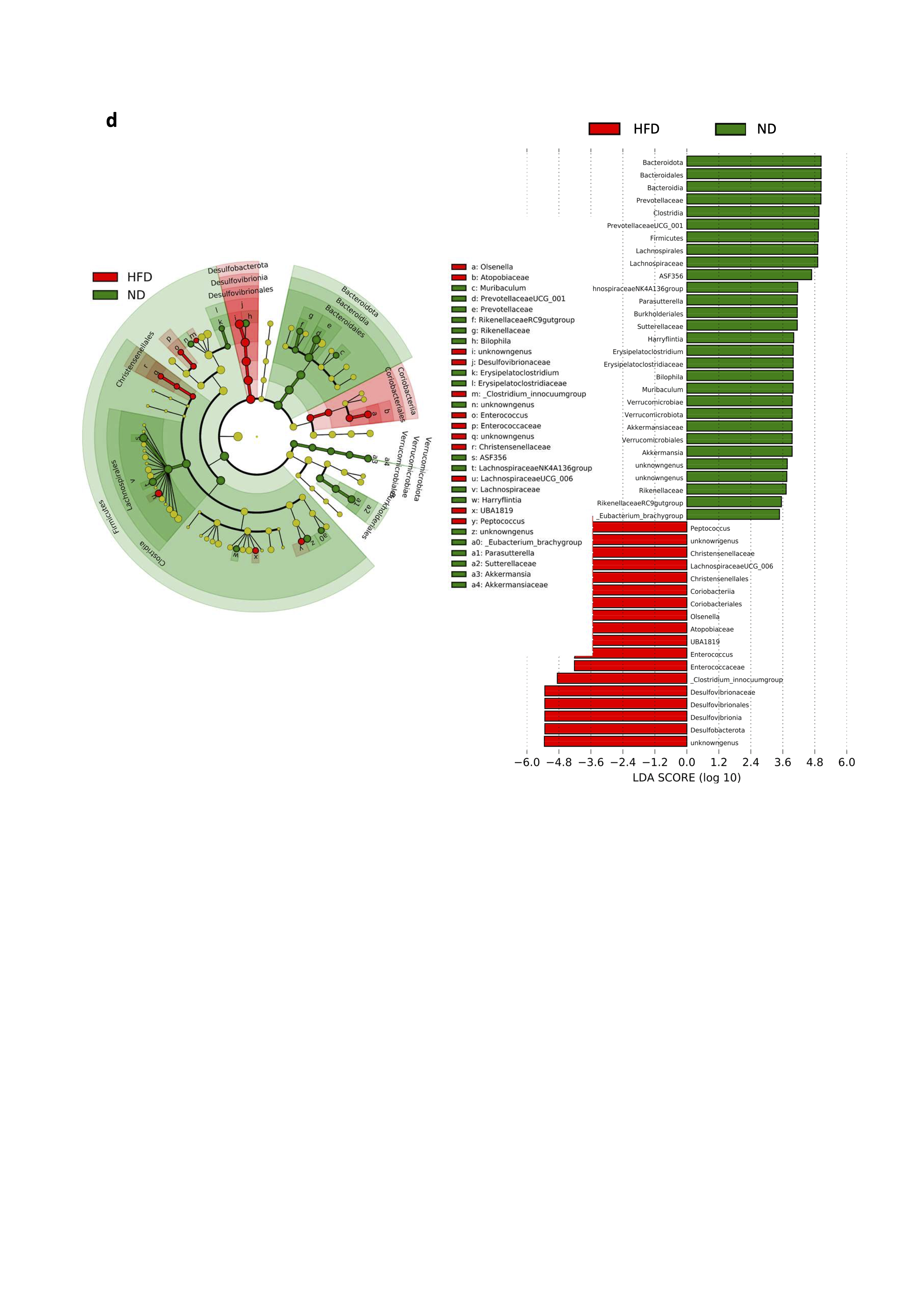
**

**Supplementary Figure S3. Study 1: fecal microbiota characterization of C57Bl/6J mice fed with either normal diet (ND) or high fat diet (HFD) after colonization with/out *Entamoeba muris (E. muris).*** **(a)** alpha-diversity indexes (Observed, Chao1 and InvSimpson). **(b)** beta-diversity indexes (Jaccard, Unifrac and Weight-Unifrac). Ellipses represent 95% of confidence. Statistical analyses were performed using PERMANOVA. **(c-d)** Taxonomic representation in a cladogram (left) and the Linear Discriminant Analysis (LDA) score (right) comparing (**c**) ND *vs*. ND + *E. muris* or (**d**) HFD *vs* ND*.* Dot plotted data presents mean ± SEM. Statistical analyses were performed using the One-way ANOVA test followed by a Bonferroni post hoc test. Significant differences were recorded as *p<0.05, **p<0.01, ***p<0.001, ****p<0.0001. Differences corresponding to P values lower than 0,01 are reported for LDA analyses.

**
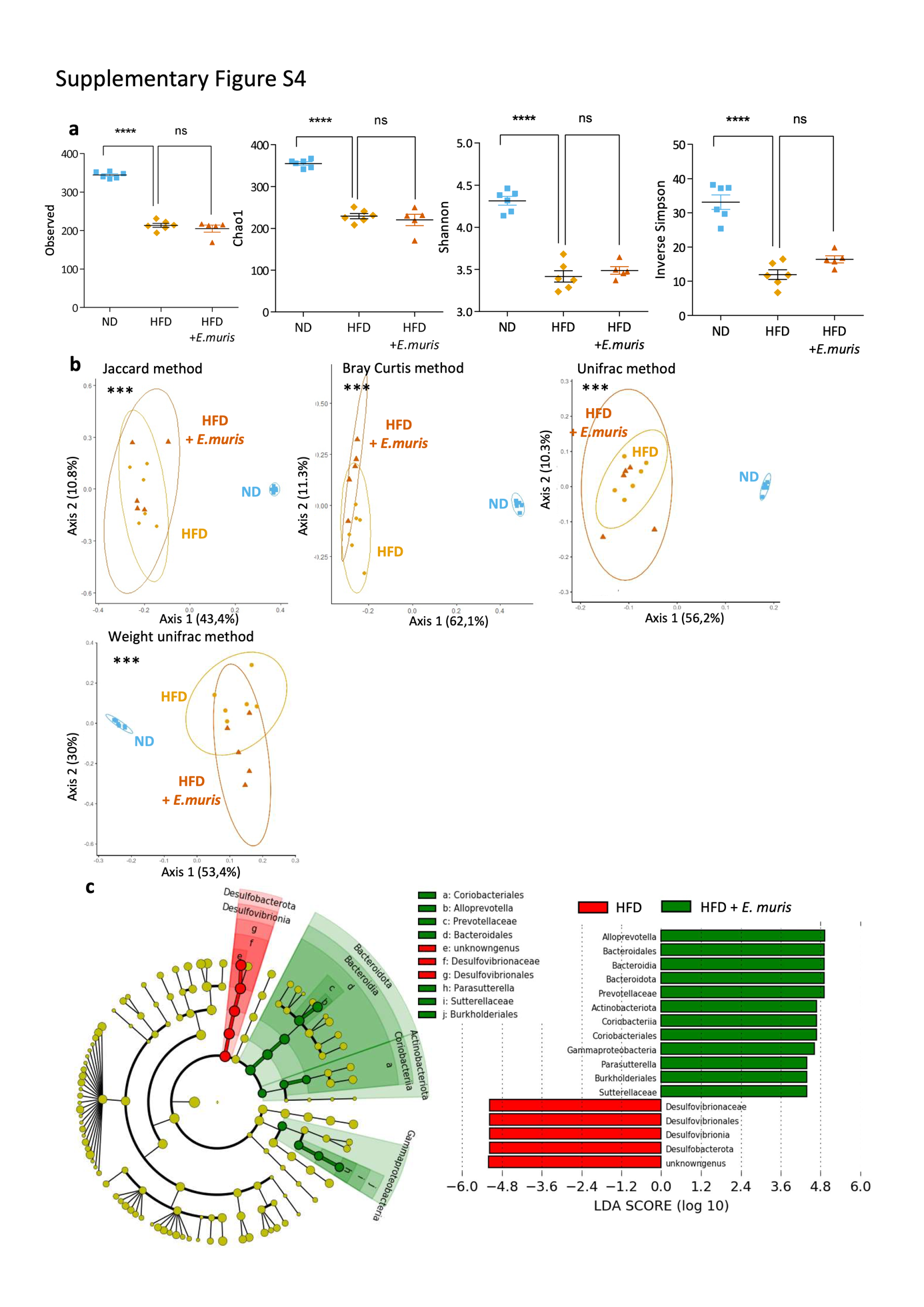

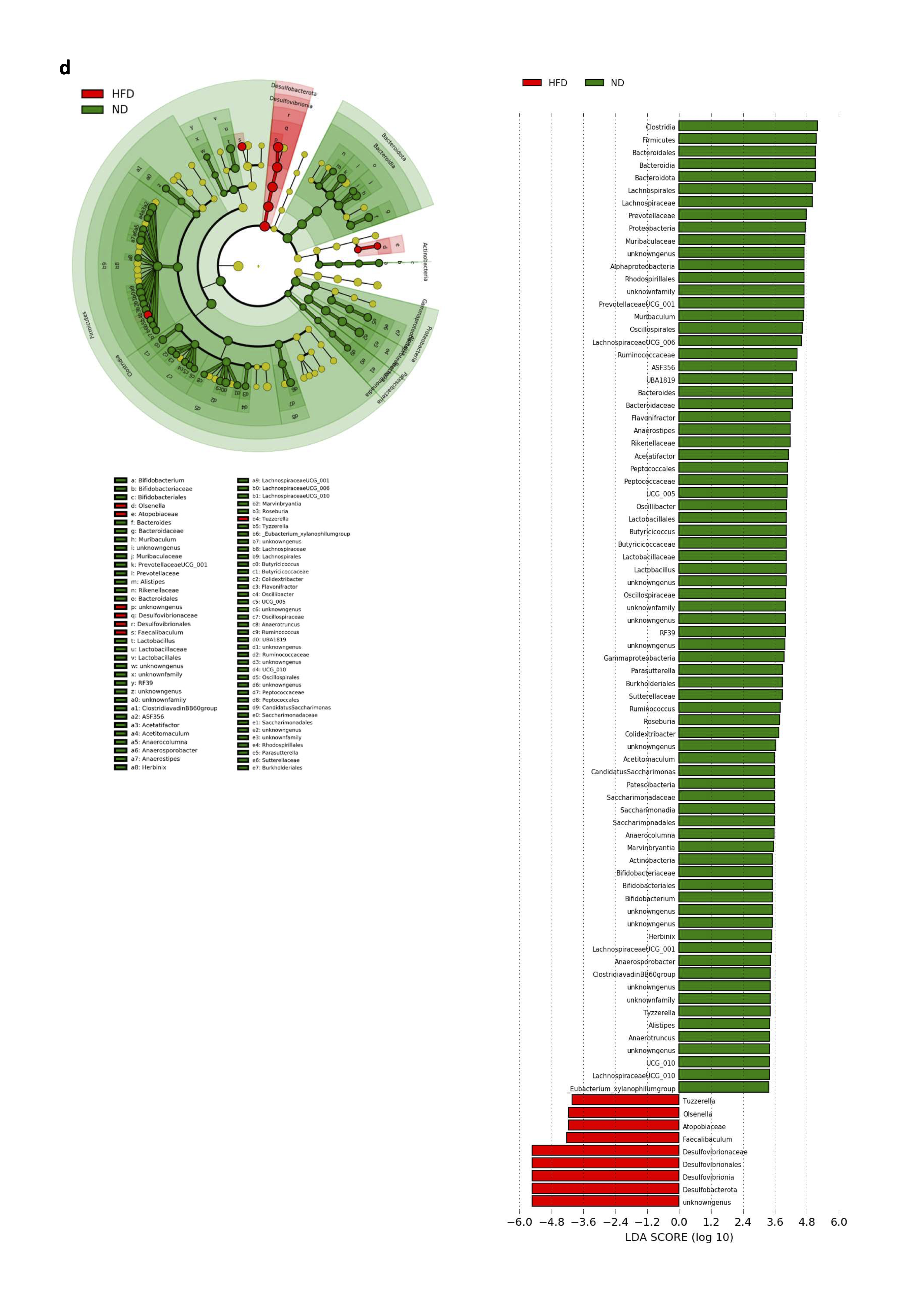
 Supplementary Figure S4. Study 2: fecal microbiota characterization of C57Bl/6J mice fed with either normal diet (ND) or high fat diet (HFD) after colonization with/out *Entamoeba muris (E. muris).*** **(a)** alpha-diversity indexes (Observed, Chao1, Shannon and InvSimpson). **(b)** beta-diversity indexes (Jaccard, Bray-Curtis, Unifrac and Weight-Unifrac). Ellipses represent 95% of confidence. Statistical analyses were performed using PERMANOVA. **(c-d)** Taxonomic representation in a cladogram (left) and the Linear Discriminant Analysis (LDA) score (right) comparing (**c**) HFD *vs*. HFD + *E. muris* or (**d**) ND *vs* HFD*.* Dot plotted data presents mean ± SEM. Statistical analyses were performed using the One-way ANOVA test followed by a Bonferroni post hoc test. Significant differences were recorded as *p<0.05, **p<0.01, ***p<0.001, ****p<0.0001. Differences corresponding to P values lower than 0,01 are reported for LDA analyses.

**
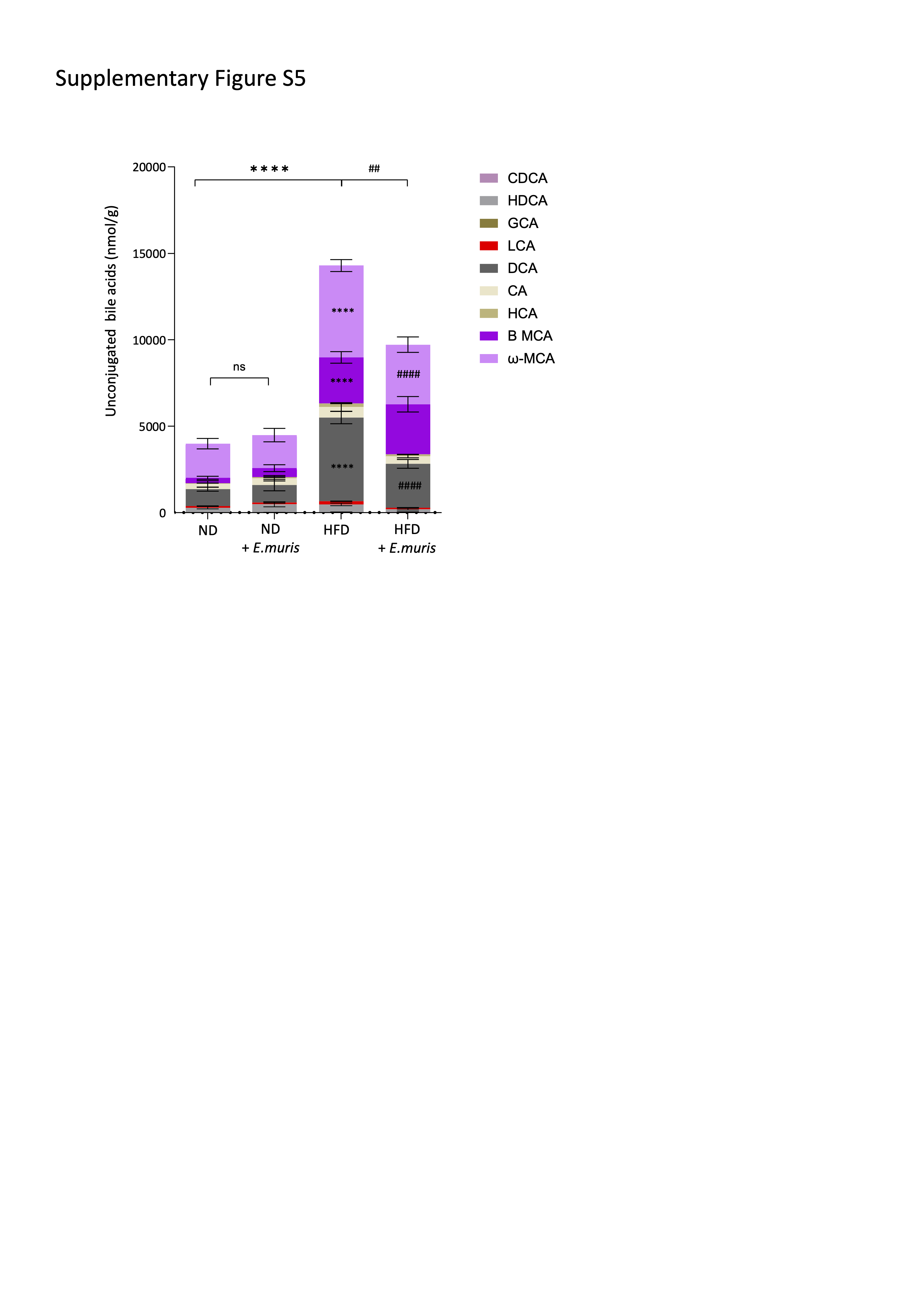
 Figure S5. Detailed analysis of unconjugated bile acids in the feces.** Concentrations of the different unconjugated bile acids are shown. Statistical analyses were performed using Mixed-effects analysis or One-way ANOVA test, both followed by a Bonferroni post hoc test. Significant differences were recorded as *p<0.05, **p<0.01, ***p<0.001, ****p<0.0001 when comparing the same bile acid type between groups, and ####p<0.0001 when comparing different bile acids in one same condition. βMCA: β-Muricholic acid, CA: Cholic acid, CDCA: Chenodeoxycholic acid, DCA: Deoxycholic acid, GCA: Glycocholic acid, HCA: Hyocholic acid, HDCA: Hyodeoxycholic acid, LCA: Lithocholic acid, ω−MCA: ω-Muricholic acid. Data were pooled from the two studies.

**
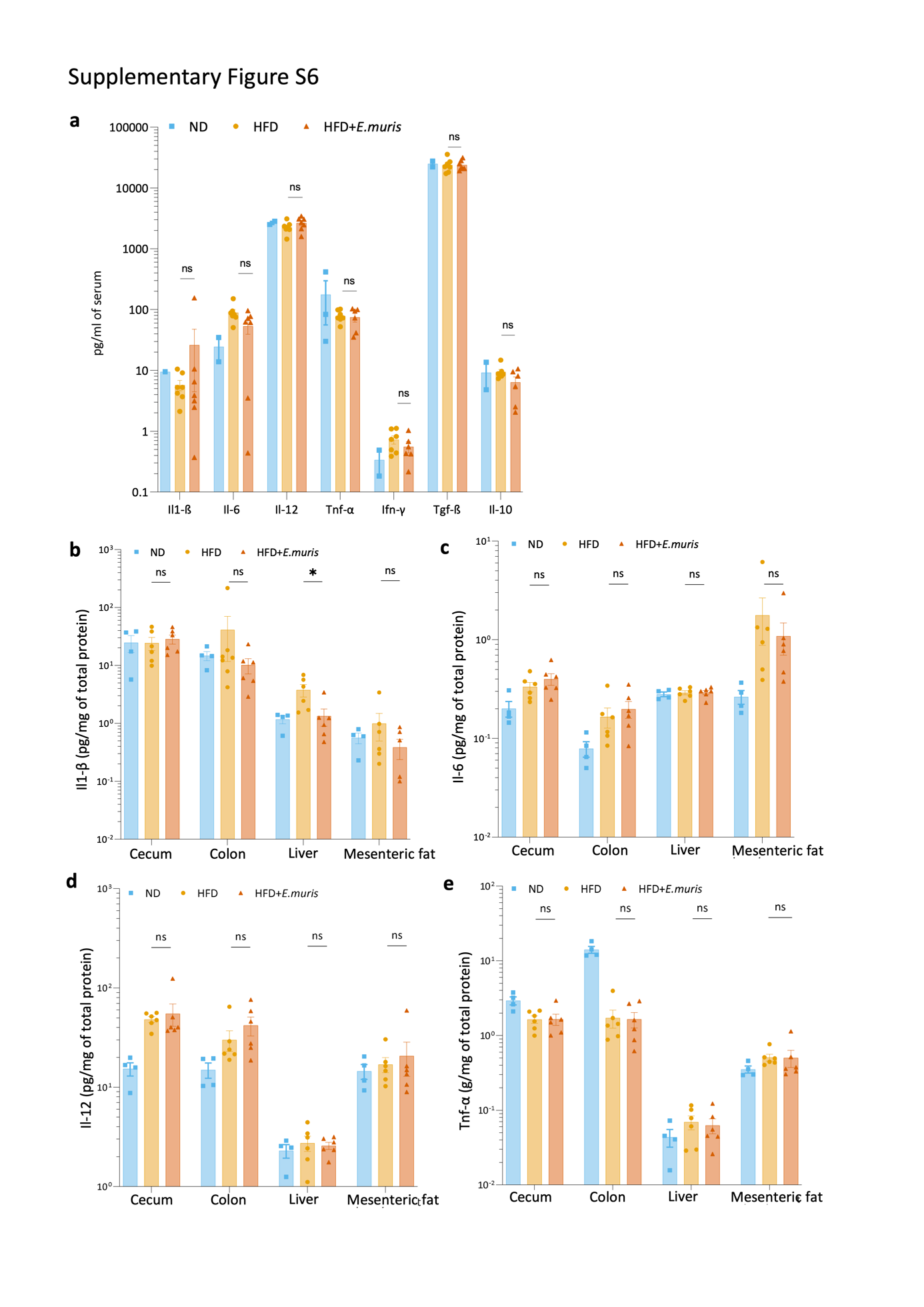
Supplementary Figure S6. Impact of E. muris in HFD-fed mice on cytokine concentrations.** Concentrations of interleukin 1β (Il-1β), interleukin 6 (Il-6), interleukin 12 (Il-12), Tumor necrosis factor α (Tnf-α), interferon γ (Ifn-γ) transforming growth factor beta (Tgf-β) and interleukin 10 (Il-10) were measured in **(a)** the serum and **(b-h)** several tissues including cecum, colon, liver and mesenteric fat. Data are presented as mean ± SEM. Statistical analyses were performed using a *t*-test comparing HFD mice with/out *E. muris*. Significant differences were recorded as *p<0.05, **p<0.01. Data were provided by study 2.


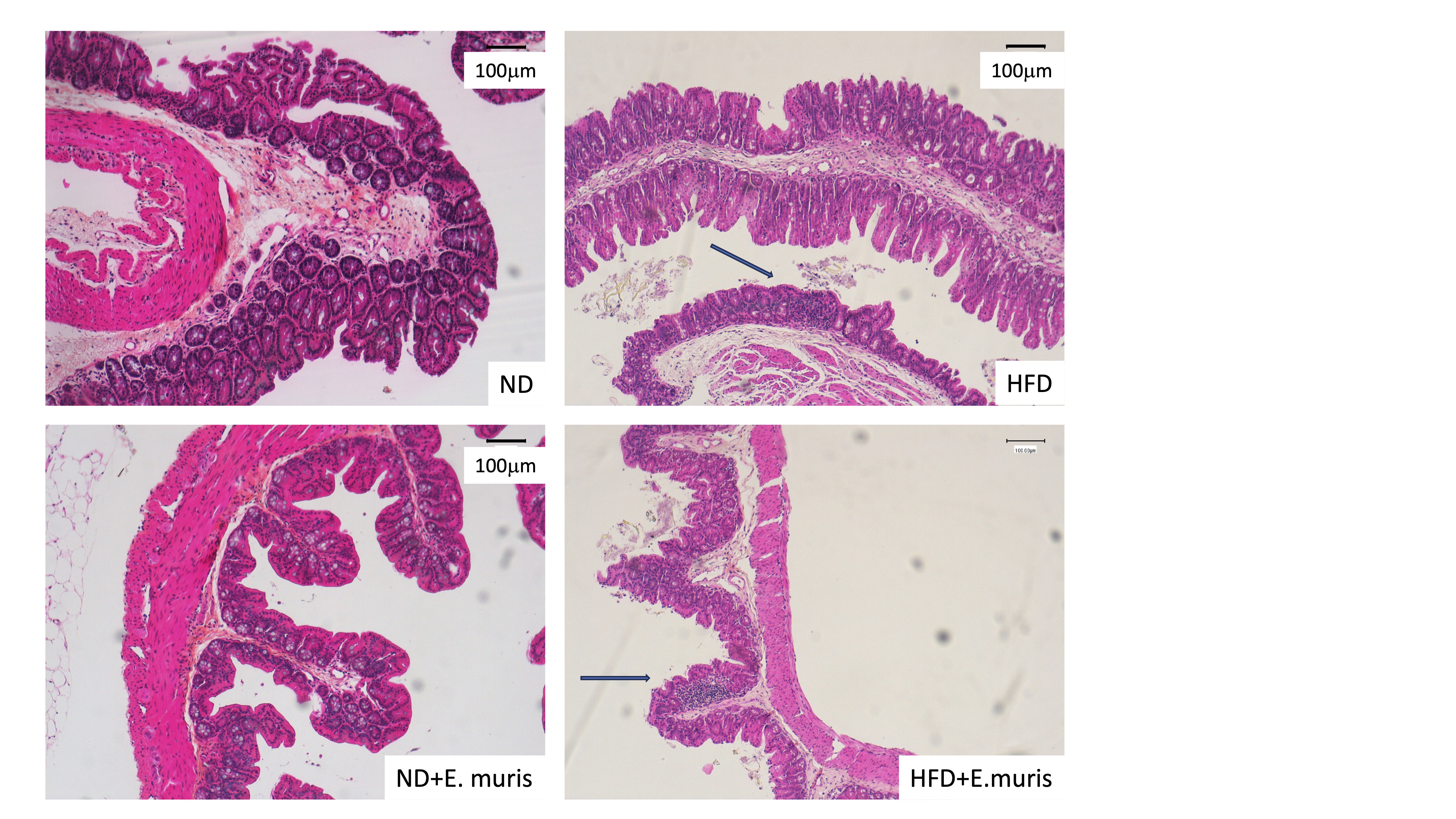


**Supplementary figure S7. Representative images of cecal histological sections from the 4 experimental groups (H&E coloration).** Arrows indicate focal inflammatory lesions in HFD mice.

**
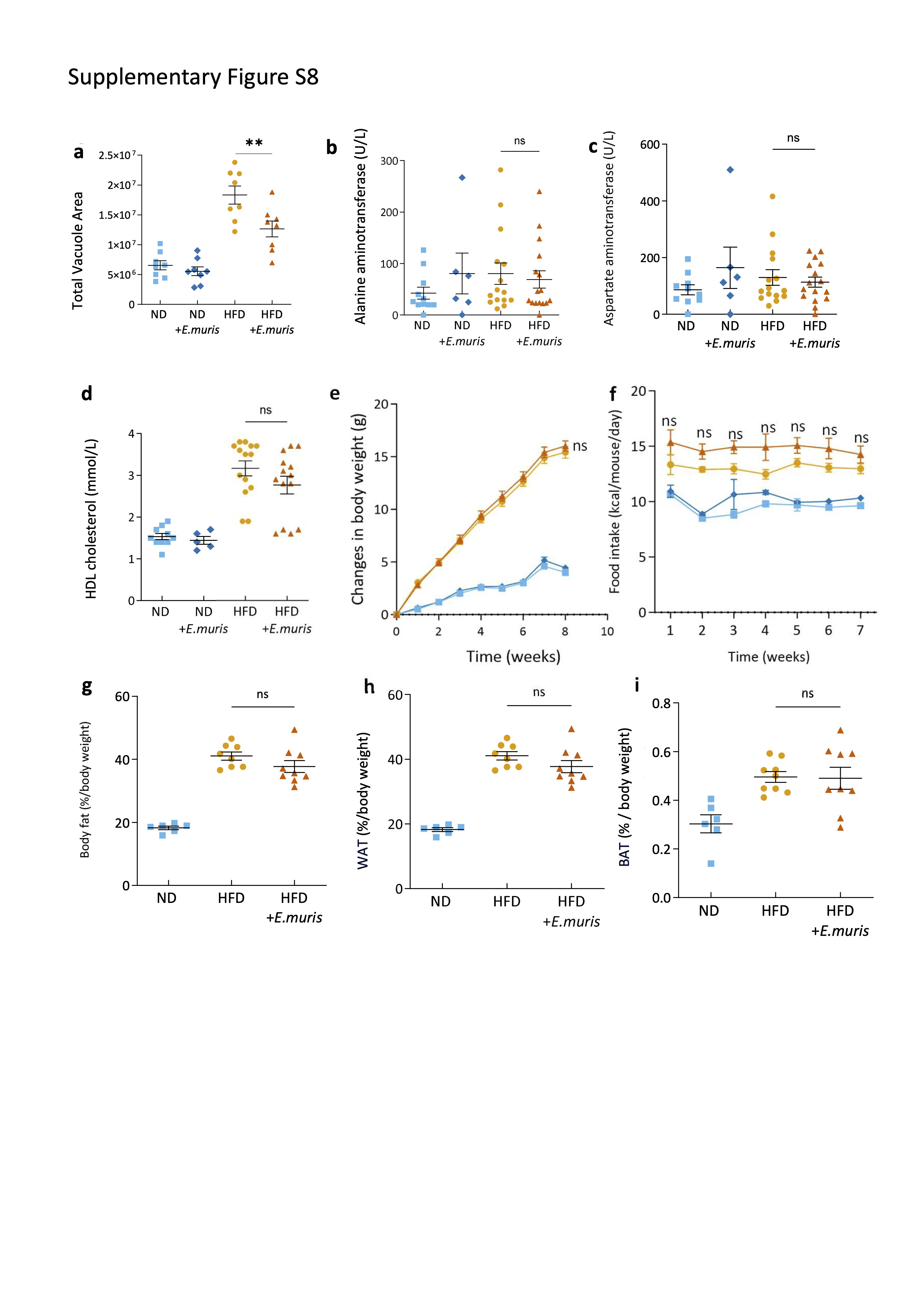
**

**
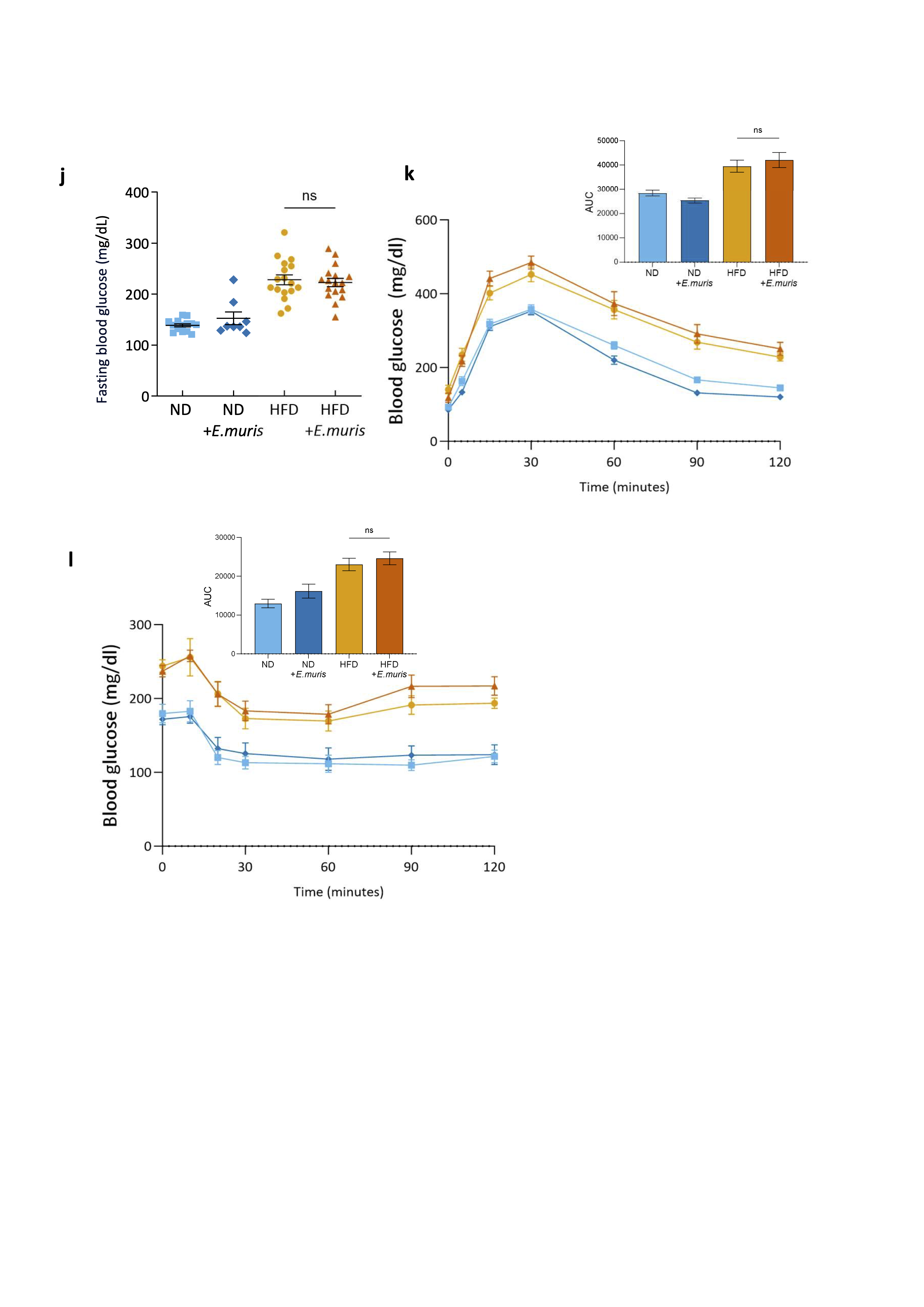
**

**Supplementary Figure S8. Impact of *E. muris* colonization on the metabolic syndrome in HFD-fed mice.** C57Bl/6J mice were fed with normal (ND) or high fat diet (HFD) and colonized with/out *Entamoeba muris* (*E. muris*). (**a**) Proportion of vacuole areas in liver slides. (**b**) Alanine and (**c**) aspartate aminotransferases were measured to evaluate the liver damage. (**d**) High-density lipoprotein (HDL) cholesterol levels in the serum. (**e**) Changes in body weight. (**f**) Mean daily food intake per day. **(g)** Body fat composition measured by bioelectrical impedance analysis. Visceral fat was evaluated by calculating the percentage of (**h**) epididymal white adipose tissue (WAT) and (**i**) brown adipose tissue (BAT) in relation to body weight. (**j**) Blood sugar levels measured after 17 hours of fasting. (**k**) Oral glucose tolerance test (**l**) insulin tolerance test. Data are presented as mean ± SEM. Statistical analyses were performed using the Mann-Whitney U test or Mixed-effects analysis followed by a Bonferroni post hoc test. Data were provided from both studies (a-f and j-l) or study 2 only (g-i).
